# 3D-printed helmet-type neuro-navigation approach (I-Helmet) for transcranial magnetic stimulation

**DOI:** 10.3389/fnins.2023.1224800

**Published:** 2023-08-07

**Authors:** He Wang, Dong Cui, Jingna Jin, Xin Wang, Ying Li, Zhipeng Liu, Tao Yin

**Affiliations:** ^1^Institute of Biomedical Engineering, Chinese Academy of Medical Science and Peking Union Medical College, Tianjin, China; ^2^School of Radiology, Shandong First Medical University and Shandong Academy of Medical Sciences, Shandong, China; ^3^Neuroscience Center, Chinese Academy of Medical Science & Peking Union Medical College, Beijing, China

**Keywords:** transcranial magnetic stimulation (TMS), coil positioning, coil orientation, landmark guide, individualized positioning, helmet-type method, clinically friendly way, I-Helmet

## Abstract

Neuro-navigation is a key technology to ensure the clinical efficacy of TMS. However, the neuro-navigation system based on positioning sensor is currently unable to be promoted and applied in clinical practice due to its time-consuming and high-cost. In the present study, we designed I-Helmet system to promote an individualized and clinically friendly neuro-navigation approach to TMS clinical application. I-Helmet system is based on C++ with a graphical user interface that allows users to design a 3D-printed helmet model for coil navigation. Besides, a dedicated coil positioning accuracy detection method was promoted based on three-dimensional (3D) printing and 3D laser scanning for evaluation. T1 images were collected from 24 subjects, and based on each image, phantom were created to simulate skin and hair. Six 3D-printed helmets with the head positioning hole enlarged by 0–5% tolerance in 1% increments were designed to evaluate the influences of skin, hair, and helmet-tolerance on the positioning accuracy and contact force of I-Helmet. Finally, I-Helmet system was evaluated by comparing its positioning accuracy with three skin hardnesses, three hair styles, three operators, and with or without landmarks. The accuracy of the proposed coil positioning accuracy detection method was about 0.30 mm in position and 0.22° in orientation. Skin and hair had significant influences on positioning accuracy (*p* < 0.0001), whereas different skin hardnesses, hair styles, and operators did not (*p* > 0.05). The tolerance of the helmet presented significant influences on positioning accuracy (*p* < 0.0001) and contact force (*p* < 0.0001). The positioning accuracy significantly increased (*p* < 0.0001) with landmark guided I-Helmet. 3D-printed helmet-type Neuro-navigation approach (I-Helmet) with 3% tolerance and landmarks met the positioning requirements for TMS in clinical practice with less than 5 N mean contact force, 3–5 mm positioning accuracy, 65.7 s mean operation time, and 50-yuan material cost. All the results suggest that the cost of I-Helmet system may be much less than the that of training clinical doctors to position the coil of TMS operation during short period of time.

## Introduction

Transcranial magnetic stimulation (TMS) has been widely applied in the treatment of clinical psychiatric diseases for over 20 years (Barker et al., 1985; Hallett, 2007; Kuo et al., 2022). Based on the principle of induction, a pulse current is driven through a stimulation coil placed on the subject’s head, and an induced current is generated on the area of the cerebral cortex located underneath the coil (Maxwell, 1865). Since the position of the coil on the subject’s head determines the functional area of the brain it stimulates, the accuracy and reliability of coil positioning provide the foundation for effective clinical treatment (Sparing et al., 2010).

To date, several approaches have been used to position the stimulation coil. The simplest coil positioning method relies on external anatomical landmarks, such as midline or ear-to-ear-line. A function-guided coil positioning method uses a hotspot at the M1 as a reference for locating other functional areas. For example, it is assumed that the premotor cortex is 2–3 cm anterior to the hotspot (Herwig et al., 2001), the dorsolateral prefrontal cortex is 5 cm anterior to the hotspot (Gerschlager et al., 2001), and the primary somatosensory cortex is 3 cm posterior to the hotspot (Koch et al., 2006). Another method is to use the 10–20 electrode system to record EEG to guide the coil positioning (Herwig et al., 2003). The above-mentioned approaches are convenient and low cost but may lead to a coil positioning error of several centimeters due to the variability of individual anatomy and function (Okamoto et al., 2004).

To improve the positioning accuracy of the stimulation coil, real-time neuro-navigation systems have been developed (Lefaucheur, 2010; Ruohonen and Karhu, 2010). Several commercial neuro-navigation systems for TMS are currently available, such as Brainsight™, developed by Rogue Research Inc., and Visor2™, developed by Advanced Neuro Technology B.V. The accuracy of neuro-navigation systems depends on the registration error. The error of the landmark registration method is around 5–6 mm (Ruohonen and Karhu, 2010). The error of the iterative closest point (ICP) registration method is around 2–3 mm, but dozens of position points need to be collected on the subject’s head (Chen and Medioni, 1991; Besl and McKay, 1992). Due to their high application cost and cumbersome operation, neuro-navigation technology is not widely used in the clinical practice of TMS (Herbsman et al., 2009; Weigand et al., 2017; Jiang et al., 2022).

In a previous study, the authors created a helmet-type coil positioning method for TMS called I-Helmet (Wang et al., 2022). I-Helmet was developed with C++, and a graphical user interface was provided to allow users to create a 3D-printed helmet model for coil positioning. Experimental evaluation showed that millimeter-level accuracy of coil positioning could be realized in under 40 s operation time. These results indicated that I-Helmet could provide an individualized and clinically friendly coil positioning approach, which is especially important in the clinical field of TMS. However, several problems need to be solved to make I-Helmet suitable for clinical application. First, in the previous study, a home-made neuro-navigation system was applied to test the coil positioning accuracy of I-Helmet. The coil calibration and registration error of the neuro-navigation system was taken as the detection error for testing the coil positioning accuracy of I-Helmet approach. At present, there is no detection method available to more accurately determine the coil positioning and orientation error of I-Helmet. Second, a 3D-printed hard head phantom was applied for experimental evaluation. In real application scenarios, the deformation of soft tissues such as scalp and hair will lead to mismatch between the subject’s head and helmet. This mismatch will produce a large contact force on the subject’s head and will also affect the coil positioning accuracy.

To solve these problems and promote the clinical application of I-Helmet, a dedicated coil positioning accuracy detection method was developed for I-Helmet and its detection error was evaluated. Next, a phantom was used to simulate real skin and hair with silicone 3D-printed skin and wigs to test the influence of skin and hair on the coil positioning accuracy and contact force of I-Helmet. Next, for each subject, six helmets were designed with the head positioning hole enlarged by 0–5% in 1% increments to enlarge the tolerance between the subject’s head and the helmet to test the influence of the tolerance on the contact force and coil positioning accuracy. Then, landmark guided I-Helmet was designed and tested using a phantom with 3D-printed skin and wig. Next, the effects of three different skin hardnesses and three different wig styles on the coil positioning accuracy of landmark guided I-Helmet were compared. Finally, two operators were recruited to test whether operators could master the I-Helmet approach with short training time.

## Methods

The details of the proposed methods can be found in the Supplementary material. A brief description is presented in this section.

### Dedicated coil positioning accuracy detection method for I-Helmet

**Figure 1:**
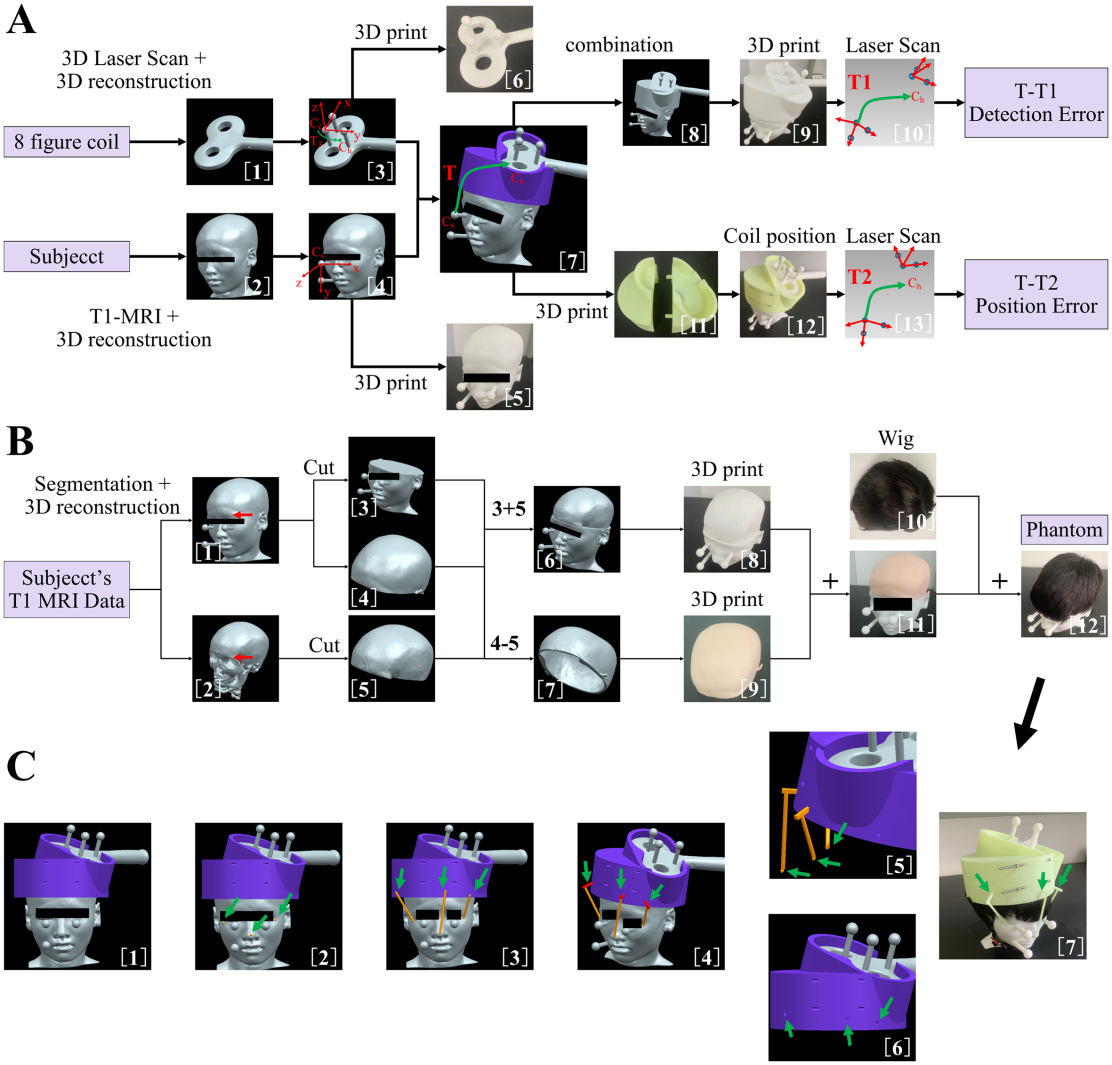
**(A)** Dedicated coil positioning accuracy detection method. (1) three-dimensional (3D) reconstruction of figure-eight coil. (2) 3D reconstruction of subject’s head. (3) Addition of three manually set spheres on figure-eight coil model. (4) Addition of three manually set spheres on subject’s head model. (5) 3D-printed subject’s head with manually set spheres. (6) 3D-printed figure-eight coil with manually set spheres. (7) Coil 3D model positioning based on the position and orientation of the target and definition of transformation matrix T. (8) Merging of coil model, helmet model, and subject’s head model. (9) 3D-printed merged 3D model. (10) Scan of 3D-printed merged 3D model and definition of transformation matrix T1. (11) 3D-printed helmet. (12) Coil positioning with helmet. (13) Scan of combined three 3D-printed models and definition of transformation matrix T2. **(B)** Method of making the phantom to simulate skin and hair. (1) Subject’s head model with manually set spheres. (2) 3D reconstruction of subject’s skull. (3) Lower part of subject’s head model. (4) Upper part of subject’s head model. (5) Upper part of subject’s skull model. (6) Construction of skull model. (7) Construction of skin model. (8) 3D-printed skull model with hard resin. (9) 3D-printed skin model with silica gel. (10) Wig used to simulate hair in the present study. (11) Combined 3D-printed skin and skull model. (12) Construction of phantom model with soft skin and hair. **(C)** (1) Subject’s head model, helmet model, and coil model in imaging space. (2) Addition of three spheres on subject’s head model. (3) Addition of three cylinders of landmarks. (4) Addition of three cuboids of the landmark and construction of three landmark sticks for helmet positioning. (5) Lower parts of the landmark sticks were processed with Boolean difference. (6) Generation of mounting holes on the helmet for landmark sticks. (7) Coil positioning with landmark guided I-Helmet.

In the present study, a dedicated coil positioning accuracy detection method was developed for I-Helmet. As shown in Figure 1A, the 3D models of the subject’s head and figure-eight coil were constructed with 3D images collected by MRI and 3D laser scan [Figure 1A (1, 2)]. Then, three 20-mm-diameter spheres were placed on each of the two models. The spherical centers of the three spheres on each model followed the rule that the three spherical centers can form two orthogonal vectors, which are defined as the *x*-axis and *y*-axis, and then, the *z*-axis can be obtained by cross multiplication of the *x*-axis and *y*-axis [Figure 1A (3, 4)]. Two coordinate systems were constructed for the coil 3D model (C_c_) and the subject’s head 3D model (C_s_). The transformation (*T_3_*) from C_c_ to the hotspot of the coil (C_h_, which is defined in ref) was obtained in the imaging space [Figure 1A (3)]. Then, the planned coil position (defined in ref) was determined based on the position and orientation of the stimulation target, and the coil 3D model was placed on the subject’s head 3D model with the coil hotspot at the planned coil position. The transformation (T) from C_s_ to C_h_ in imaging space was calculated, and C_h_ was set as the planned coil position [Figure 1A (7)]. Next, the coil model, helmet model, and subject model were merged together [Figure 1A (8)] and 3D printed [Figure 1A (9)]. Finally, the merged 3D model was 3D scanned, and the spherical centers of the six spheres were collected [Figure 1A (10)]. Correspondingly, two coordinate systems were constructed for the coil 3D model (C_c_^’^) and the subject’s head 3D model (C_s_^′^) in laser scan space, and the transformation (T1) from C_s_^′^ to C_h_^’^ was calculated. Because of the error of the 3D printing and 3D laser scanning, T and T1 will not be equal. Thus, the detection error of the proposed coil positioning accuracy detection method can be obtained from the difference between T and T1.

Then, the coil model, helmet model, and subject’s head model were 3D printed separately [Figure 1A (5, 6, 11)] and assembled following the I-Helmet coil positioning method [Figure 1A (12)]. Next, the combined 3D model was 3D scanned, and the spherical centers of the six spheres were collected [Figure 1A (13)]. Similarly, two coordinate systems were constructed for the coil 3D model (C_c_^”^) and the subject’s head 3D model (C_s_^″^) in laser scan space, and the transformation (T2) from C_s_^″^ to the C_h_^”^ was calculated. Because of the error of installation and mechanical matching error between three 3D-printed models, T and T2 will not be equal; thus, the coil positioning error can be obtained from the difference between T and T2. The mathematical principle of the proposed method is derived from the hand-eye calibration technology of the TMS robot (Lars et al., 2011; Wang et al., 2018). See the Supplementary material (Supplementary Figure S1) for detailed formula derivation of the coil positioning and orientation errors in this study.

### The method of making phantom to simulate skin and hair

To test the influences of skin and hair on the positioning accuracy and contact force of I-Helmet, a phantom was made to simulate skin and hair with silicone 3D-printed skin and a wig. As shown in Figure 1B, the 3D model of the subject’s skull was reconstructed from structural MRI data [Figure 1B (2)]. Then, the 3D models of the subject’s head and skull were cut into upper and lower parts along the eyebrow arch [Figure 1B (3–5)]. Next, the lower part of the subject’s head model and the upper part of the subject’s skull model were subjected to a Boolean operation of sum to create a skull model [Figure 1B (6)]. Simultaneously, the upper part of the subject’s head and the upper part of the subject’s skull were subjected to a Boolean operation of difference to create a skin model [Figure 1B (7)]. The skull model was 3D printed with hard resin [Figure 1B (8)], and the skin model was 3D printed using soft silicone to simulate the hardness of skin [Figure 1B (9)]. Then, the skin model was placed on the skull model to create a test model with skin [Figure 1B (11)]. Finally, a wig was put on the skin model to create a simulated phantom to test the influences of skin and hair on the positioning accuracy and contact force of I-Helmet [Figure 1B (12)].

### Manufacturing and application of landmark guided I-Helmet

To reduce the influences of skin and hair on the accuracy of I-Helmet, a landmark guided approach was developed in this study based on landmark registration, which is applied in traditional neuro-navigation systems. As shown in Figure 1C, three round balls with a diameter of 6 mm each were placed at the left and right corners of the eyes and the tip of the nose [Figure 1C (2)]. A cylinder was generated in the helmet direction with each spherical center as the origin [Figure 1C (3)]. The diameter of the cylinder was 5 mm, and the center of the cylinder’s lower surface was located at the center of the ball just generated. The upper surface of the cylinder was above the lower surface of the helmet, and the cylinder did not intersect the helmet. Three cuboids were generated on the upper surface of the three cylinders [Figure 1C (4)]. The direction of the longest side of the cuboid was perpendicular to the direction of the cylinder height. The cuboids had to intersect the helmet and be more than 10 mm deep into the helmet, and they could not penetrate the helmet. The three spheres, cylinders, and cuboids were subjected to a Boolean sum operation to produce three landmark sticks. The landmark sticks and the head model of the subject were subjected to a Boolean operation of difference to generate the shape of the head model of the subject under the landmark sticks [Figure 1C (5)]. The helmet and the landmark sticks were subjected to a Boolean operation of difference to generate mounting holes on the helmet for the landmark sticks [Figure 1C (6)]. Finally, the landmark sticks and helmet were 3D printed separately, and the landmark sticks were mounted in their own mounting holes on the helmet and put on the subject’s head with the guidance of the images [Figure 1C (7)].

## Results

### Detection error analysis for proposed coil positioning accuracy detection approach for I-Helmet

**Figure 2:**
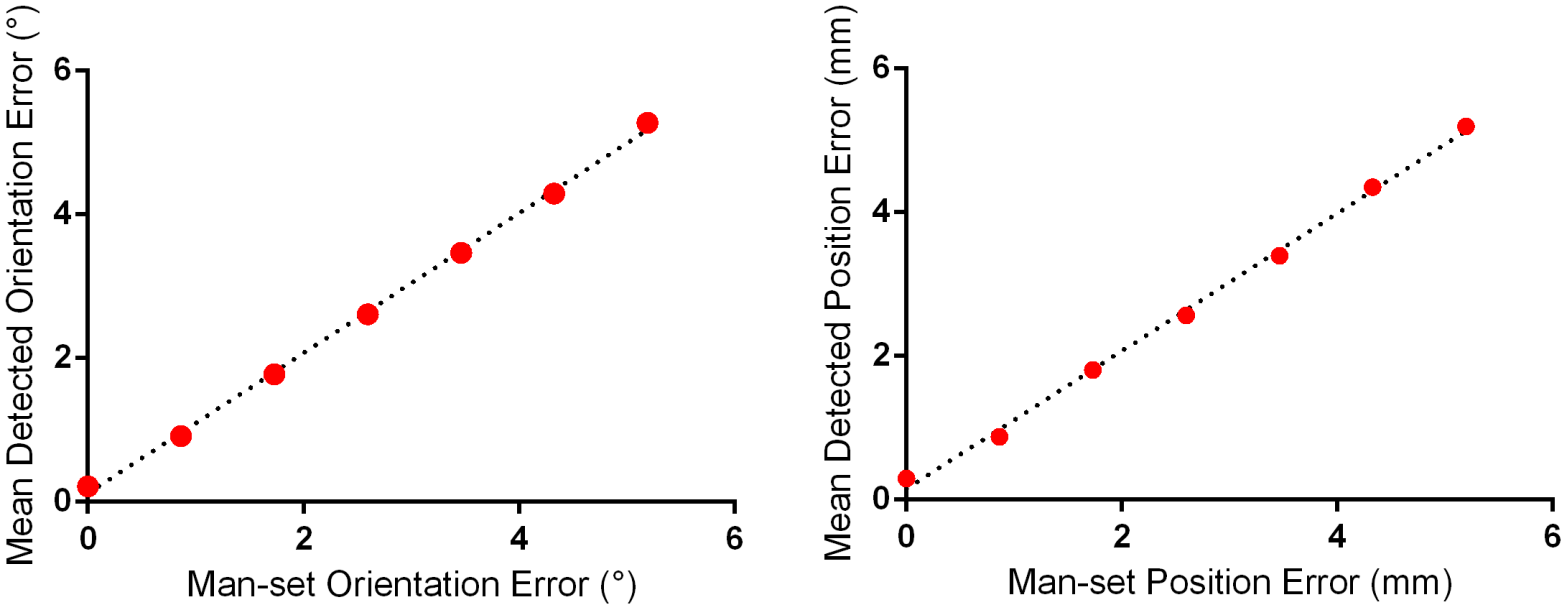
Detection error evaluation for proposed positioning accuracy detection approach for I-Helmet. The mean detected position and orientation errors were determined with 10 repeated 3D scans based on the merged 3D models of subjects 1 and 2.

The effects of each manually set position and orientation error on the detected position and orientation error of 10 repeated 3D scans based on the merged 3D models of subjects 1 and 2 are illustrated in Figure 2. The linear regression demonstrated that the mean detected position error was significantly positively correlated with the manually set position error (*R* = 0.9974, *F* = 1944, *p* < 0.0001), and the mean detected orientation error was significantly positively correlated with the manually set orientation error (*R* = 0.9987, *F* = 3,911, *p* < 0.0001). It should be noted that the mean detected position error and orientation error were 0.30 mm and 0.22°, respectively, when the manually set position error and orientation error were both zero; this was caused by the errors of 3D printing and laser scanning.

### The influences of skin, hair, and helmet tolerance on the coil positioning accuracy and contact force of I-Helmet

**Figure 3:**
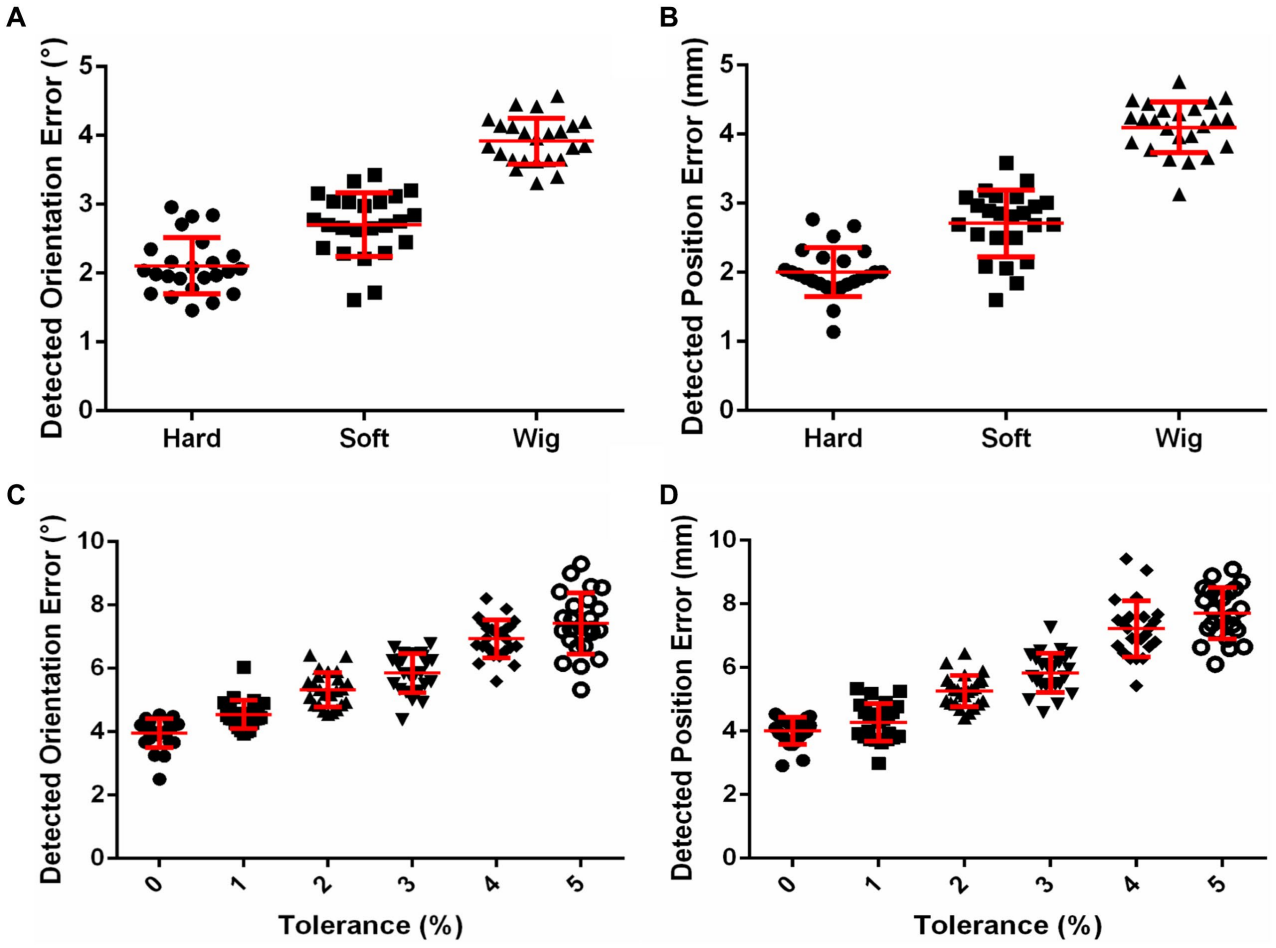
**(A)** Effects of skin and hair on coil positioning accuracy. **(B)** Effects of helmet tolerance on coil positioning accuracy.

The influences of skin and hair on the detected position and orientation error are shown in Figures 3A,B, respectively. The position errors detected with hard resin, skin, and hair phantom were 2.00 ± 0.35, 2.71 ± 0.48, and 4.1 ± 0.37 mm, respectively. The orientation errors detected with hard resin, skin, and hair phantom were 2.11 ± 0.41°, 2.71 ± 0.46°, and 3.92 ± 0.34°, respectively. One-way analysis of variance (ANOVA) indicated significant influences of model patterns on position error [*F* = 165.7, *p* < 0.0001, sum-of-squares (SS) = 65.92] and orientation error (*F* = 123.9, *p* < 0.0001, SS = 52.22). Tukey’s multiple comparison test showed that the post-hoc comparison on position and orientation errors was significant (*p* < 0.0001) for any two groups. The influences of helmet tolerance on detected position and orientation error are shown in Figures 3C,D, respectively. The mean detected position errors (defined in Supplementary Equation S16) were 4.01, 4.28, 5.26, 5.83, 7.22, and 7.70 mm as the tolerance of the helmet increased from 0 to 5% in 1% increments. The mean detected rotation errors (defined in Supplementary Equation S17) were 3.95°, 4.54°, 5.33°, 5.85°, 6.94°, and 7.42° as the tolerance of the helmet increased from 0 to 5%. One-way ANOVA indicated significant influences of helmet tolerance on position error (*F* = 125.7, *p* < 0.0001, SS = 334.1) and orientation error (*F* = 109.3, *p* < 0.0001, SS = 271.6). The influence of helmet tolerance on contact force is shown in Supplementary Figure S2. Two-way ANOVA indicated a significant influence of helmet tolerance on contact force [*F* (5, 1,104) = 2,333, *p* < 0.0001, SS = 13,001], but there was no significant difference [*F* (7, 1,104) = 0.9245, *p* = 0.4863, SS = 36.07] between the contact force detected by the eight force sensors. The means and standard deviations of contact force detected by eight force sensors on 24 phantoms were 21.34 ± 2.78, 16.69 ± 2.9, 11.77 ± 3.05, 4.73 ± 2.01, 2.11 ± 1.49, and 1.43 ± 1.3 N, respectively, for the 0, 1, 2, 3, 4, and 5% tolerance helmets.

### Effects of using helmet with or without landmarks on coil positioning accuracy

**Figure 4:**
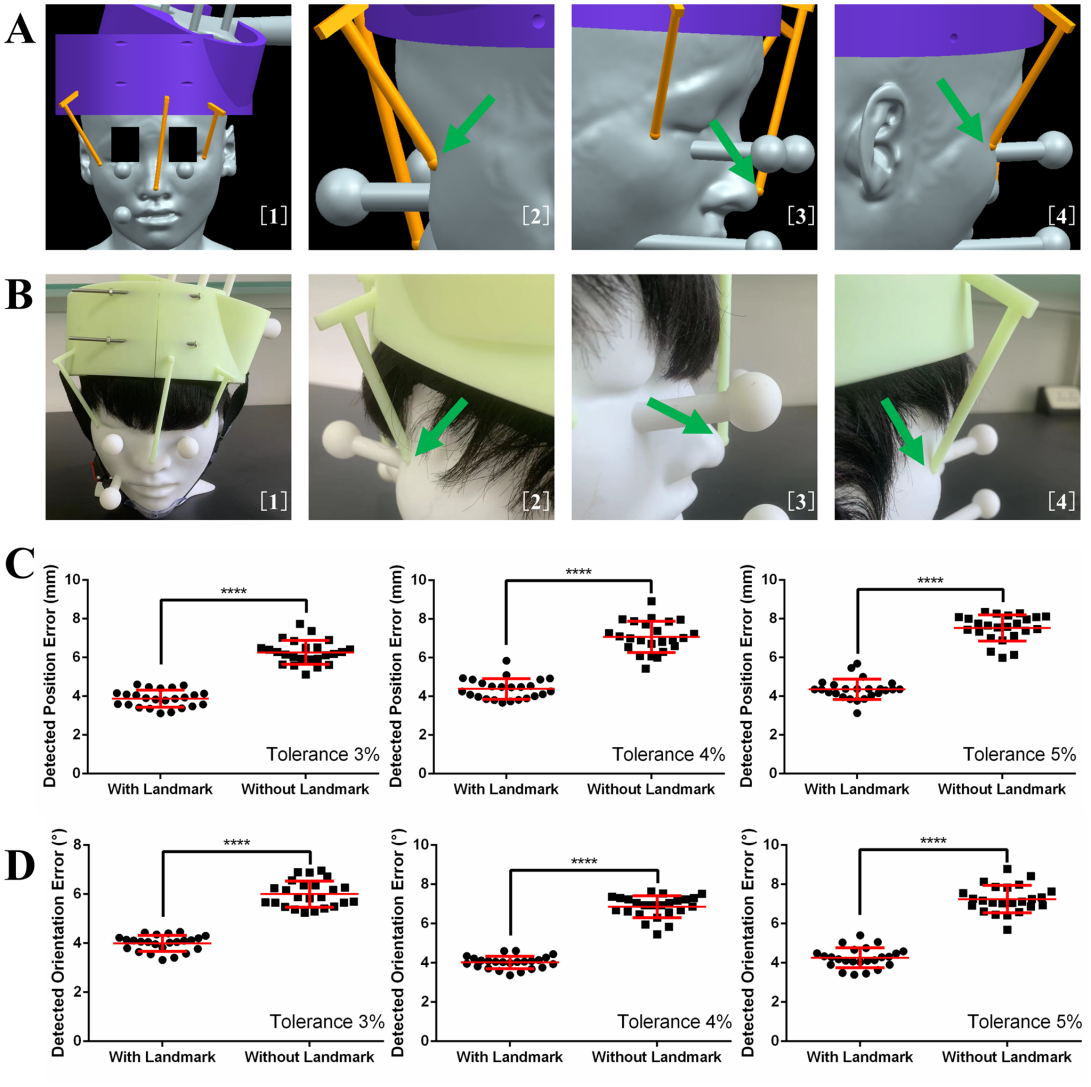
**(A)** Image guidance for landmark guided helmet. **(B)** Helmet and coil positioning with image guidance. **(C)** Detected position errors with and without landmarks. **(D)** Detected orientation errors with and without landmarks.

The three landmarks in 3D image space are shown in Figure 4A. With the guidance of these images, the helmet was put on the phantom, as shown in Figure 4B. The effects of using the helmet with or without landmarks on the translation error and rotation error are shown in Figures 4C,D, respectively. The means and standard deviations of the position error without landmarks were 6.27 ± 0.61, 7.07 ± 0.81, and 7.53 ± 0.68 mm for the 3, 4, and 5% tolerance helmets, respectively. With landmarks, the position errors were reduced to 3.88 ± 0.44, 4.39 ± 0.53, and 4.35 ± 0.53 mm for the 3, 4, and 5% tolerance helmets, respectively. The means and standard deviations of orientation error without landmarks were 6.00 ± 0.54°, 6.86 ± 0.56°, and 7.25 ± 0.70° for the 3, 4, and 5% tolerance helmets, respectively. With landmarks, the orientation errors were reduced to 4.00 ± 0.32°, 4.02 ± 0.32°, and 4.29 ± 0.51° for the 3, 4, and 5% tolerance helmets, respectively. Paired T-tests indicated that the position and orientation errors were both significantly reduced (*p* < 0.0001) by landmark guidance for the 3, 4, and 5% tolerance helmets.

### Effects of different skin hardnesses, wig styles, and operators on coil positioning accuracy of landmark guided I-Helmet

**Figure 5:**
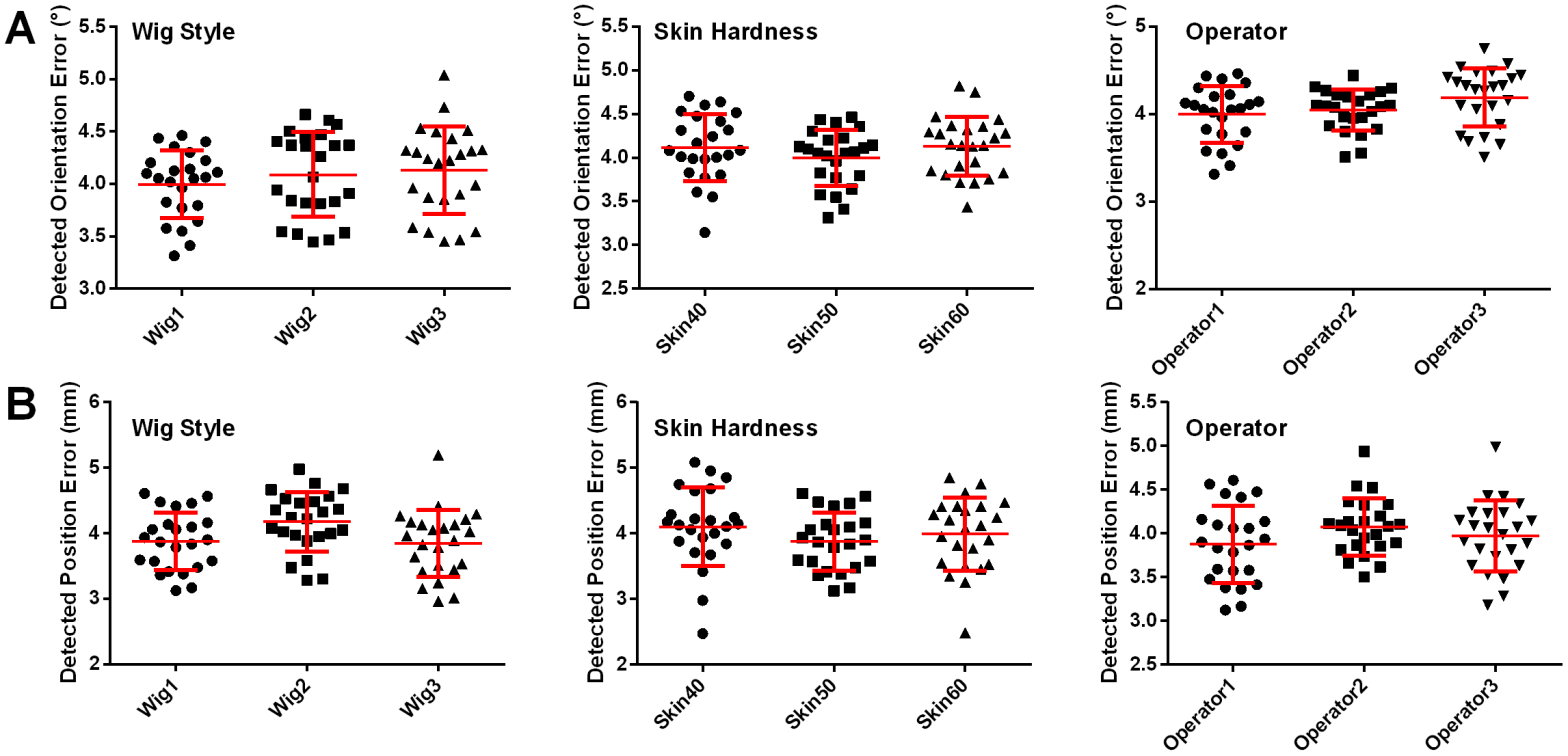
**(A)** Detected orientation errors with three different skin hardnesses, three wig styles, and three operators. **(B)** Detected position errors with three different skin hardnesses, three wig styles, and three operators.

The effects of different skin hardnesses, wig styles (Supplementary Figure S3), and operators on the position and orientation errors of coil positioning are illustrated in Figures 5A,B, respectively. One-way ANOVA indicated no significant changes (*p* = 0.0649) in detected position error among three different wig styles (wig1: 3.88 ± 0.44 mm, wig2: 4.18 ± 0.45 mm, wig3: 3.85 ± 0.51 mm) and no significant changes (*p* = 0.1083) in detected orientation error among three different wig styles (wig1: 4.00 ± 0.32°, wig2: 4.09 ± 0.41°, wig3: 4.13 ± 0.42°). One-way ANOVA indicated no significant changes (*p* = 0.2919) in detected position error among three different skin hardnesses (skin40: 4.10 ± 0.6 mm, skin50: 3.88 ± 0.44 mm, and skin60: 3.99 ± 0.56 mm) and no significant changes (*p* = 0.3340) in detected orientation error among three different skin hardnesses (skin40: 4.12 ± 0.39°, skin50: 4.00 ± 0.32°, and skin60: 4.13 ± 0.38°). One-way ANOVA indicated no significant changes (*p* = 0.2211) in detected position error among three different operators (operator1: 3.88 ± 0.44 mm, operator2: 4.08 ± 0.33 mm, and operator3: 3.97 ± 0.40 mm) and no significant changes (*p* = 0.0714) in detected orientation error among three operators (operator1: 4.00 ± 0.32°, operator2: 4.04 ± 0.23°, and operator3: 4.19 ± 0.33°). The mean execution time for the three operators was 65.7 s across 72 total trials of the coil positioning evaluation experiment with landmark guided I-Helmet approach.

## Discussion

A clinically friendly coil positioning approach for TMS is currently in demand (Lars et al., 2011; Caulfield et al., 2022). Hence, this study focused on reducing the impacts of skin and hair on the coil positioning accuracy and head contact force of I-Helmet. This paper systematically introduces a more practical landmark guided helmet-type coil positioning approach, presents a dedicated coil positioning accuracy detection method, and evaluates the accuracy of the landmark guided approach in real application scenarios. The experimental evaluation results reveal several important benefits of the landmark guided I-Helmet: first, it can accurately locate the magnetic stimulation field of TMS to the individual image space of the subject’s brain; second, it is low cost and convenient; and, third, it is an easy-to-master coil positioning method.

The currently used neuro-navigation technologies apply a tracking sensor to realize accurate coil positioning. The accuracy of neuro-navigation technology depends on the registration approach used to determine the conversion relationship between the positioning sensor coordinate system and the subject image coordinate system. The accuracy of neuro-navigation technology is reported at 2–3 mm with the ICP registration method and at 5–6 mm with the landmark registration method (Chen and Medioni, 1991; Besl and McKay, 1992; Lefaucheur, 2010; Ruohonen and Karhu, 2010). The two registration approaches minimize the average distance of two point sets collected from the subject 3D image and the tracking sensor after registration (Chetverikov et al., 2005; Wang and Fang, 2009; Du et al., 2010; Badran et al., 2020). The minimum average distance between two point sets after registration is defined as registration error, but this kind of registration error does not actually represent the coil position and orientation error relative to the planned coil position. Under the guidance of neuro-navigation technology, the hand-held coil positioning process and coil calibration process will also affect the coil positioning accuracy (Herwig et al., 2001; Sparing et al., 2010). Besides, the registration processes of neuro-navigation systems lack a clear definition of coil orientation error. Landmark guided I-Helmet can realize the accurate positioning of the coil without positioning sensors; so, we promoted an error detection method without positioning sensors in present study. Regarding the coil positioning accuracy of landmark guided I-Helmet, the position and orientation errors mostly ranged from 3 to 5 mm and from 3° to 5°, respectively. In contrast to traditional neuro-navigation technology, the position and orientation errors of coil positioning can be clearly defined and detected with landmark guided I-Helmet. Therefore, the coil positioning accuracy of landmark guided I-Helmet meets the needs of TMS in clinical practice.

In this study, a coil positioning accuracy detection method was proposed and demonstrated to have detection errors of 0.30 mm in terms of position and 0.22° in terms of orientation. The position detection error was similar to that of the currently used tracking sensor (the widely used near-infrared binocular vision positioning sensor Polaris Vega ST has a positioning accuracy of 0.3 mm with a 95% confidence interval). Additionally, skin and hair were shown to significantly affect the accuracy of I-Helmet. Supplementary Figure S4A shows that because I-Helmet applies mechanical cooperation to realize coil positioning, the mechanical mismatch between the head and helmet caused by skin and hair will inevitably lead to the reduction of coil positioning accuracy. The mechanical mismatch will also lead to a contact force exceeding 20 N. Previous TMS robot studies have shown that the comfortable head contact force is around 5 N (Banerjee et al., 1995; Zorn et al., 2012). Considering that the helmet is 3D-printed with hard resin and the treatment time of rTMS is typically about 20 min, a contact force exceeding 20 N will cause significant discomfort to the helmet wearer (Gershon et al., 2003; Rossi et al., 2009; Richter et al., 2012). To reduce the contact force, the head positioning hole of the helmet was magnified by 1–5%. As the size of the head positioning hole increased, the head contact force decreased and the coil positioning accuracy also decreased. Supplementary Figures S4C,D helps explain these results, showing that the contact force was caused by the gravity of the helmet and the mechanical squeezing; so, as the head positioning hole on the helmet enlarged, the squeeze on the head decreased, the mismatch between the head and the helmet increased, and the coil positioning accuracy decreased. The experiments showed that it was most appropriate to enlarge the head positioning hole of the helmet by 3%, because the head had an acceptable contact force. When the head positioning hole was enlarged to 4% or 5%, the helmet became relatively unstable.

To improve the coil positioning accuracy, three landmark sticks were designed to guide helmet positioning. This design refers to the landmark registration approach of traditional neuro-navigation systems (Chetverikov et al., 2005; Ruohonen and Karhu, 2010). The results presented in Figure 4 show that the landmark can significantly improve the coil positioning accuracy. In addition, considering that the low weight of the landmark does not significantly increase the material cost and the installation step does not significantly increase the operation difficulty, the landmark guided I-Helmet approach is very suitable for TMS clinical practice. Besides, Figure 5 shows that the operation of landmark guided I-Helmet is very easy to master. Two operators without any experience in TMS or neuro-navigation approaches were able to fully master the operation of landmark guided I-Helmet through training lasting less than 10 min.

In terms of convenience and cost, traditional neuro-navigation systems require a series of complicated operations such as image processing, registration, and coil calibration, and commercially available neuro-navigation systems for TMS are high-cost instruments. Based on the software system architecture mode of client and server of I-Helmet, which is detailed in Supplementary Figure S3A of reference (Wang et al., 2022), for each patient, the neurologist only needs to perform three steps to achieve accurate coil positioning: upload patient 3D images and target information, place the 3D-printed helmet on the patient’s head with the guidance of landmark sticks, and put the stimulation coil in the coil positioning hole on the helmet. To the best of our knowledge, landmark guided I-Helmet is the most time-saving coil positioning approach with millimeter-level positioning accuracy. The time–cost of the 10–20 system is about 16 min, as reported in reference (Kobayashi and Pascual-Leone, 2003 Xiao et al., 2017). The time–cost of scalp-measurement based parameter space is about 4.4 min, as reported in reference (Jiang et al., 2022). In the present study, the mean execution time of I-Helmet by three operators on 24 phantoms was 65.7 s.

This study was conceived to provide a landmark guided I-Helmet coil positioning method for TMS. All the results supported that landmark guided I-Helmet is a convenient, low-cost, and easy-to-master method suitable for TMS clinical practice. The potential applications of landmark guided I-Helmet suggested in the present study should be methodologically and clinically verified in future research. Applied research on the use of the I-Helmet for brain stimulation utilizing other physical fields such as transcranial ultrasound stimulation will also be carried out in the future.

## Conclusion

TMS has been certified by the FDA for treating several clinical diseases such as depression. In this study, a 3D-printed coil positioning method was developed for TMS, and it appeared to solve the limitations of currently applied methods in terms of convenience, cost, and accuracy. Evaluation results with a 3D-printed phantom covered in silicone skin and a wig indicated that the positioning accuracy, execution time, and material cost of the landmark guided I-Helmet with 3% tolerance meet the coil positioning requirements of TMS clinical practice. I-Helmet may be a highly efficient training model for doctors to grasp the positioning skill and improve the experience of target schedule of TMS.

## Data availability statement

The original contributions presented in the study are included in the article/Supplementary material, further inquiries can be directed to the corresponding authors.

## Ethics statement

The studies involving human participants were reviewed and approved by the Joint Ethics Committee of the Tianjin Normal University and the Institute of Biomedical Engineering, the Chinese Academy of Medical Sciences, and Peking Union Medical College. The patients/participants provided their written informed consent to participate in this study.

## Author contributions

HW: conceptualization, investigation, funding acquisition, methodology, project administration, visualization, writing – original draft, and writing – review and editing. TY: conceptualization, investigation, and writing – review and editing. ZL: project administration, resources, and writing – review and editing. JJ: investigation. DC and XW: data curation. YL: conceptualization and writing – review and editing. All authors contributed to the article and approved the submitted version.

## Funding

ZL acknowledges grant support from the Natural Science Foundation of China (Grant No. 81927806). Funding to pay the Open Access publication charges for this article was provided by the CAMS Initiative for Innovative Medicine (2021-I2M-1-015, recipient HW) and the Natural Science Foundation of China (Grant No. 81801798). TY acknowledges grant support from the CAMS Initiative for Innovative Medicine (2021-I2M-1-058). HW acknowledges grant support from the Non-profit Central Research Institute Fund of Chinese Academy of Medical Sciences (2020-JKCS-006).

## Conflict of interest

The authors declare that the research was conducted in the absence of any commercial or financial relationships that could be construed as a potential conflict of interest.

## Publisher’s note

All claims expressed in this article are solely those of the authors and do not necessarily represent those of their affiliated organizations, or those of the publisher, the editors and the reviewers. Any product that may be evaluated in this article, or claim that may be made by its manufacturer, is not guaranteed or endorsed by the publisher.
