# Supplementary material for: 3D-printed helmet-type neuro-navigation approach (I-Helmet) for transcranial magnetic stimulation
Source: Front Neurosci. 2023 Aug 7;17:1224800. doi: 10.3389/fnins.2023.1224800 (PMC10442160; doi:10.3389/fnins.2023.1224800)
Supplement: Supplementary file 1 [file Data_Sheet_1.docx]

# Landmark guided helmet-type coil positioning approach (I-Helmet) for transcranial magnetic stimulation Supplementary Materials

**He Wang^1*^, Dong Cui^3^, Jingna Jin^1^, Xin Wang^1^, Ying Li^1^, Zhipeng Liu^1*^, Tao Yin^1,2*^**

^1^Institute of Biomedical Engineering, Chinese Academy of Medical Science & Peking Union Medical College, Tianjin 300192, China

^2^Neuroscience Center, Chinese Academy of Medical Science & Peking Union Medical College, Beijing 100730, China

^3^Department of Radiology, The Second Affiliated Hospital of Shandong First Medical University, Shandong, China

*** Correspondence:**

He W, PhD.

whe19882006@126.com

Zhipeng L, Ph.D.
[lzpeng67@163.com](mailto:lzpeng67@163.com)

Tao Y, Ph.D.
[bme500@163.com](mailto:bme500@163.com)

1. **Subjects**

Structural T1 MRI images from 24 healthy volunteers (12 young adults, aged 19–24 years, 6 females and 6 males; 12 older adults, aged 49–80 years, 6 females and 6 males) were used for experimental evaluations in this study. None of the subjects reported discomfort or other reactions during the study.

1. **Image Collection**

MRI data of young adults were collected at the Tianjin Normal University using a Siemens 3T Prisma scanner (Siemens, Erlangen, Germany) with a 32-channel head coil. The following parameters were applied to acquire structural T1 images: MPRAGE T1-weighted scans, TR = 2300 ms, TE = 2.25 ms, voxel size = 1 mm^3^, FOV = 256 mm × 256 mm, flip angle = 9°, 176 sagittal slices.

MRI data of older adults were collected on matched 3T Trio MR scanners (Siemens, Erlangen, Germany) using the 12-channel phased array head coil at the second affiliated hospital of Shandong First Medical University. Foam padding was used to minimize head motion for all subjects. Structural MRI data were obtained using a sagittal magnetization-prepared rapid gradient echo (MPRAGE) three-dimensional T1-weighted sequence with the following imaging parameters: MPRAGE T1-weighted scans, TR = 6.1 ms, TE = 2.0 ms, voxel size = 1 mm^3^, FOV = 256 mm × 256 mm, flip angle = 9°, 176 sagittal slices.

A 3D laser scan system (RigelScan Max, Hexagon Manufacturing Intelligence Co., Ltd., QingDao, China) was used for scanning a stimulation figure-eight coil (70 mm figure-eight coil, Magstim, Whitland, UK) and spheres in the coil positioning scene.

1. **Image Preprocessing**

The T1 images for each subject were segmented into scalp and gray matter with a neuro-navigation software package (BrainSight, London, UK). Skulls were segmented from T1 images with the Statistical Parametric Mapping (SPM) 12 software package (London, UK) in MATLAB 2018a (MathWorks, Natick, MA, USA). Finally, the segmented scalp and gray matter images were imported into the I-Helmet software system, and the 3D model of the head and brain was displayed in the global coordinate system of the individual image space based on the isosurface reconstruction algorithm of VTK (Visualization Toolkit, Version 5.10.1), as shown in Figure 1A(1). The 3D model display of the figure-eight coil was realized based on the 3D point cloud reconstruction algorithm of VTK and stored in files of STL format, as shown in Figure 1A(2).

1. **Setup of the Spheres on the 3D Model of Subject’s Head and Figure-Eight Coil**

As shown in Figure 1A(3) and Supplementary Figure 1(1), three 20-mm-diameter spheres were put on the 3D model of the figure-eight coil and defined as spheres A, B, and C. The three spheres were located on the top of the 3D model of the figure-eight coil. The centers of the three spheres were each about 50 mm away from the 3D model of the figure-eight coil. The three spheres and the figure-eight coil were connected with a 10-mm-diameter cylinder. The centers of spheres A and B were 50 mm apart, and the centers of spheres A and C were also 50 mm apart.

As shown in Figure 1A(4) and Supplementary Figure 1(4), three 20-mm-diameter spheres were put on the 3D model of the subject’s head and defined as spheres D, E, and F. Spheres D and E were located below the subject's eyes at both sides of the nose, and sphere F was located below sphere D and was 50 mm from sphere D. Spheres D and E were 70 mm apart. The centers of the three spheres were about 50 mm away from the 3D model of the subject’s head. The three spheres and the subject’s head were connected with a 10-mm-diameter cylinder.

The STL data of the subject’s head and figure-eight coil were imported into the I-Helmet software system. After the above settings were completed, the 3D models of the subject’s head and figure-eight coil with spheres were restored to STL format files and 3D-printed, as shown in Figure 1A(5) and (6).

1. **Helmet Design for Coil Positioning with I-Helmet System**

Detailed information on the modeling process of I-Helmet can be found in the second section of the supplementary martials of supplementary reference 1. Briefly, as shown in Figure 1A(7), first, the stimulation target was set on the 3D model of the subject’s brain. Second, the planned coil position and orientation were determined based on the stimulation target. Third, the hotspot coordinate system C_h_ on the coil 3D model was determined with the position and orientation of the maximum output magnetic field on the stimulation coil. Fourth, the coil 3D model was put on the subject’s head 3D model with the coil hotspot at the planned coil position. Based on the current positions of these 3D models, a positioning helmet 3D model was created.

In previous studies, in the final step of helmet design, the head 3D model was subtracted from the helmet model to create a head-shaped hole to fix the helmet on the subject's head. In the present study, for each subject, six helmets were fabricated with the head 3D model enlarged by 0%, 1%, 2%, 3%, 4%, and 5% in the last step of helmet design to enlarge the tolerance between the subject’s head and the helmet. This enabled the influence of the tolerance on the contact force and coil positioning accuracy to be tested.

1. **Determination of Coordinate Transformation (T) in Imaging Space**

Supplementary Figure 1(1)–(6) shows the c**oordinate transformation (T) in imaging space**. As shown in Supplementary Figure 1(1), the coordinates of the centers of three spheres (A, B, C) were defined as (*X_A_, Y_A_, Z_A_*), (*X_B_, Y_B_, Z_B_*), and (*X_C_, Y_C_, Z_C_*), respectively, in imaging space. So, the origin (o_coil_) and three axes (x_coil_, y_coil_, and z_coil_) of the sphere-determined coordinate system on coil C_c_ could be defined with a 4 × 4 pose matrix and expressed as follows:

$C_{c}=\left[ x_{\mathrm{coil}},y_{\mathrm{coil}},z_{\mathrm{coil}},o_{\mathrm{coil}} \right],$ (S1)

where

$$\begin{matrix} \begin{matrix} o_{coil}=\left( X_{A},Y_{A},Z_{A},1 \right)^{T}; \\ x_{coil}=\vec{AB}=\left( X_{B}-X_{A},{Y_{B}-Y}_{A},{Z_{B}-Z}_{A},0 \right)^{T}; \end{matrix} \\ \begin{matrix} y_{coil}=\vec{AC}=\left( X_{C}-X_{A},{Y_{C}-Y}_{A},{Z_{C}-Z}_{A},0 \right)^{T}; \\ z_{coil}={(\vec{AB}\times\vec{AC})}^{T}. \end{matrix} \end{matrix}$$

The transformation T3 from C_c_ to C_h_ can be determined directly from imaging space. As shown in Supplementary Figure 1(4), the coordinates of the centers of three spheres (D, E, F) are defined as (*X_D_, Y_D_, Z_D_*), (*X_E_, Y_E_, Z_E_*), and (*X_F_, Y_F_, Z_F_*), respectively, in imaging space. So, the origin (*o_head_*) and three axes (*x_head_*, *y_head_*, and *z_head_*) of the sphere-determined coordinate system on the subject’s head C_s_ can be defined with a 4 × 4 pose matrix and expressed as follows:

$C_{s}=\left[ x_{\mathrm{head}},y_{\mathrm{head}},z_{\mathrm{head}},o_{\mathrm{head}} \right],$ (S2)

where

$$\begin{matrix} \begin{matrix} o_{\mathrm{head}}=\left( X_{D},Y_{D},Z_{D},1 \right)^{T}; \\ x_{\mathrm{head}}=\vec{DE}=\left( X_{E}-X_{D},{Y_{E}-Y}_{D},{Z_{E}-Z}_{D},0 \right)^{T}; \end{matrix} \\ \begin{matrix} y_{\mathrm{head}}=\vec{DF}=\left( X_{F}-X_{D},{Y_{F}-Y}_{D},{Z_{F}-Z}_{D},0 \right)^{T}; \\ z_{\mathrm{head}}={(\vec{DE}\times\vec{DF})}^{T}. \end{matrix} \end{matrix}$$

Then, the transformation T4 from C_s_ to C_c_ in imaging space can be expressed as follows:

$T4={C_{s}}^{-1}*C_{c}.$ (S3)

Because the coil 3D model was put on the subject’s head model based on the position and orientation of the stimulation target, the relative position of the coil 3D model and head 3D model shown in Figure 1A(7) is the planning coil position for stimulating the corresponding target. Finally, we can obtain the transformation *T*, which is shown in Figure 1A(7), from C_s_ to the planned coil position in imaging space as follows:

$T=T4*T3,$ (S4)

where *T* represents the imaging-space transformation from the sphere-determined coordinate system on the subject’s head to the planned coil position when stimulating the setup target on the subject’s brain.


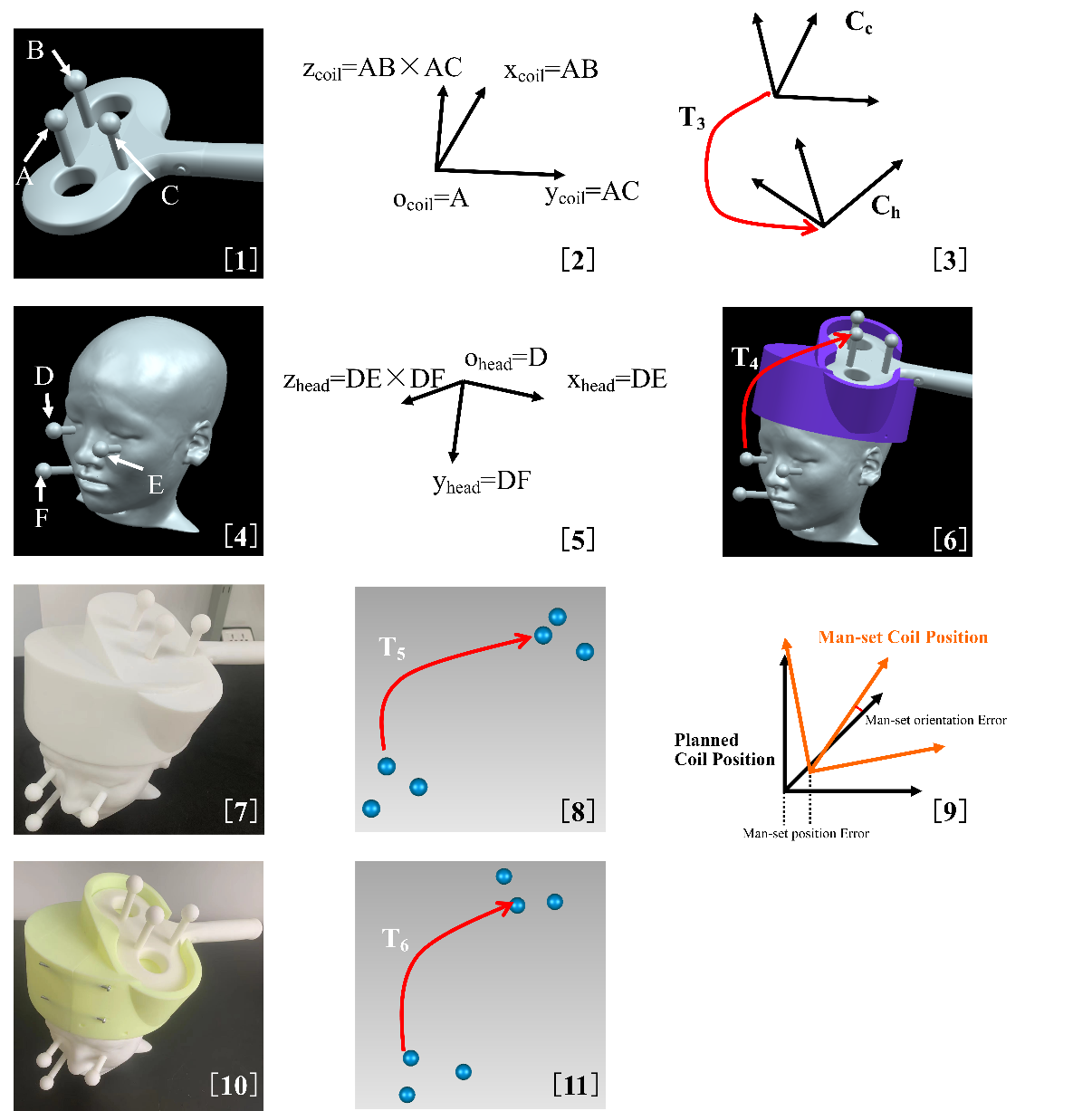


**Supplementary Figure 1.** (1) Addition of three spheres on figure-eight coil model. (2) Definition of sphere-determined coordinate system (C_c_) on figure-eight coil model. (3) Transformation (T3) from C_c_ to hotspot of figure-eight coil (C_h_). (4) Addition of three spheres on subject’s head model. (5) Definition of sphere-determined coordinate system (C_s_) on subject’s head model. (6) Transformation (T4) from C_s_ to C_c_. (7) 3D-printed merged 3D model. (8) Six spheres collected by 3D-scanning the merged model and transformation (T5) from C_s_’ to C_c_’ in laser scan space. (9) Diagram of manually set position and orientation errors. (10) Coil positioning with I-Helmet. (11) Six spheres collected by 3D-scanning the combined 3D model and transformation (T6) from C_s_’’ to C_c_’’ in laser scan space.

1. **Determination of the detection error by comparing the difference of coordinate transformation in laser-scan space (T1) and image space (T)** **with merged 3D model**

Figure 1A(8)–(10) and Supplementary Figure1(7)–(9) show the detection error definition by comparing the difference coordinate transformation in laser-scan space and image space with the merged 3D model. As shown in Figure 1A(8), a Boolean sum operation was performed on the coil 3D model, helmet 3D model, and head 3D model to obtain the merged 3D model. The merged model was 3D-printed, as shown in Figure 1A(9) and Supplementary Figure 1(7). Next, the merged 3D-printed model was scanned with a 3D laser scanner, and the centers of six spheres on the merged model were obtained, as shown in Supplementary Figure 1(8). The coordinates of the centers of three spheres (A’, B’, C’) on the coil were defined as (*X_A_*’*, Y_A_*’*, Z_A_*’), (*X_B_*’*, Y_B_*’*, Z_B_*’), and (*X_C_*’*, Y_C_*’*, Z_C_*’), respectively. So, the origin (o_coil_’) and three axes (x_coil_’, y_coil_’, and z_coil_’) of the sphere-determined coordinate system on the coil C_c_’ could be defined with a 4 × 4 pose matrix and expressed as follows:

$C_{c}’=\left[ x_{\mathrm{coil}}’,y_{\mathrm{coil}}’,z_{\mathrm{coil}}’,o_{\mathrm{coil}}’ \right]^{\perp},$ (S5)

where

$$\begin{matrix} \begin{matrix} o_{coil}’=\left( X_{A}’,Y_{A}’,Z_{A}’,1 \right)^{T}; \\ x_{coil}’=\vec{A’B’}=\left( X_{B}’-X_{A}’,{Y_{B}’-Y}_{A}’,{Z_{B}’-Z}_{A}’,0 \right)^{T}; \end{matrix} \\ \begin{matrix} y_{coil}’=\vec{A’C’}=\left( X_{C}’-X_{A}’,{Y_{C}’-Y}_{A}’,{Z_{C}’-Z}_{A}’,0 \right)^{T}; \\ \begin{matrix} z_{coil}’=\left( \vec{A’B’}\times\vec{A’C’} \right)^{T}; \\ \end{matrix} \end{matrix} \end{matrix}$$

and$\perp$represents the orthonormalization of the matrix with the singular value decomposition (SVD) method [2-3].

The coordinates of the centers of three spheres (D’, E’, F’) on the subject’s head were defined as (*X_D_*’*, Y_D_*’*, Z_D_*’), (*X_E_*’*, Y_E_*’*, Z_E_*’), and (*X_F_*’*, Y_F_*’*, Z_F_*’), respectively. So, the origin (*o_head_*’) and three axes (*x_head_*’, *y_head_*’, and *z_head_*’) of the sphere-determined coordinate system on the subject’s head C_s_’ could be expressed as follows:

$C_{s}’=\left[ x_{\mathrm{head}}’,y_{\mathrm{head}}’,z_{\mathrm{head}}’,o_{\mathrm{head}}’ \right]^{\perp},$ (S6)

where

$$\begin{matrix} \begin{matrix} o_{\mathrm{head}}’=\left( X_{D}’,Y_{D}’,Z_{D}’,1 \right)^{T}; \\ x_{\mathrm{head}}’=\vec{D’E’}=\left( X_{E}’-X_{D}’,{Y_{E}’-Y}_{D}’,{Z_{E}’-Z}_{D}’,0 \right)^{T}; \end{matrix} \\ \begin{matrix} y_{\mathrm{head}}’=\vec{D’F’}=\left( X_{F}’-X_{D}’,{Y_{F}’-Y}_{D}’,{Z_{F}’-Z}_{D}’,0 \right)^{T}; \\ z_{\mathrm{head}}’={(\vec{D’E’}\times\vec{D’F’})}^{T}. \end{matrix} \end{matrix}$$

Then, as shown in Supplementary Figure 1(8), the transformation matrix T5 from C_s_’ to C_c_’ in laser-scan space could be expressed as follows:

$T5={C_{s}’}^{-1}*C_{c}’.$ (S7)

Finally, the transformation T1, which is shown in Figure 1A(12), from C_s_’ to the planned coil position in laser-scan space could be expressed as follows:

$T1=T5*T3.$ (S8)

T1 represents the detected transformation from the sphere-determined coordinate system on the subject’s head to the planned coil position in the merged 3D model. Ideally, T should be equal to T1 if the coil 3D model is at the planned coil position. However, because 3D printing and scanning will have errors, we can express the coil position error as follows (refer to the formula derivation in supplementary reference 2):

$e_{\mathrm{trans}}=\sqrt{{(T_{1,4}-{T1}_{1,4})}^{2}+{(T_{2,4}-{T1}_{2,4})}^{2}+{(T_{3,4}-{T1}_{3,4})}^{2}}$. (S9)

The coil orientation error could be determined as follows:

$e_{\theta}=\left| \theta\right|$, (S10)

where (k, θ) is the axis-angle representation of the rotation matrix of A, which is given by (refer to the formula derivation in supplementary reference 2)

$A=T*{T1}^{-1}$. (S11)

Imaging data of subjects 1 and 2 were applied to test the detection error of the proposed approach. To test the detection error of the positioning accuracy detection method presented in this study, a series of position and orientation errors were manually set by moving the coil 3D model to seven different positions located away from the planned coil position. The translation and rotation parameters from the planned coil position to the manually set coil position are presented in Supplementary Table 1. The translation and rotation parameters, which were defined in Supplementary Figure 1(9), respectively correspond to the manually set position and orientation errors of the TMS coil. A Boolean sum operation was performed on the coil 3D model, helmet 3D model, and head 3D model to obtain seven merged 3D models for each subject. Finally, each merged 3D model was 3D-printed and scanned 10 times independently, and the detected position and orientation errors were calculated with the equations presented in this section.

Supplementary Table 1. Translation and rotation parameters of manually set coil positions

| Stated Translation Error | | | Stated Rotation Error | | | | Total Translation Error (mm) | Total Rotation Error (°) |
| --- | --- | --- | --- | --- | --- | --- | --- | --- |
| Tx (mm) | Ty (mm) | Tz (mm) | | Rx (°) | Ry (°) | Rz (°) |  |  |
| 0 | 0 | 0 | | 0 | 0 | 0 | 0 | 0 |
| 0.5 | 0.5 | 0.5 | | 0.5 | 0.5 | 0.5 | $0.5*\sqrt{3}$ | $0.5*\sqrt{3}$ |
| 1 | 1 | 1 | | 1 | 1 | 1 | $1*\sqrt{3}$ | $1*\sqrt{3}$ |
| 1.5 | 1.5 | 1.5 | | 1.5 | 1.5 | 1.5 | $1.5*\sqrt{3}$ | $1.5*\sqrt{3}$ |
| 2 | 2 | 2 | | 2 | 2 | 2 | $2*\sqrt{3}$ | $2*\sqrt{3}$ |
| 2.5 | 2.5 | 2.5 | | 2.5 | 2.5 | 2.5 | $2.5*\sqrt{3}$ | $2.5*\sqrt{3}$ |
| 3 | 3 | 3 | | 3 | 3 | 3 | $3*\sqrt{3}$ | $3*\sqrt{3}$ |

1. **Determination of the coil position and orientation errors by comparing the difference of coordinate transformation in laser-scan space (T2) and image space (T)**

Figure 1A(11)–(13) and Supplementary Figure 1(10)–(11) show the coil position and orientation errors by comparing the difference coordinate transformation in laser-scan space and image space with separated 3D models. As shown in Figure 1A(5), (6), and (11), the 3D models of the subject’s head, the figure-eight coil, and the helmet were 3D-printed separately. Then, the coil was put on the subject’s head with the guidance of the positioning helmet. Next, the combined three 3D-printed models were scanned with a 3D laser scanner, and the centers of six spheres on the coil and subject’s head 3D model were obtained, as shown in Supplementary Figure 1(11). The coordinates of the centers of three spheres (A’’, B’’, C’’) on the coil were defined as (*X_A_*’’*, Y_A_*’’*, Z_A_*’’), (*X_B_*’’*, Y_B_*’’*, Z_B_*’’), and (*X_C_*’’*, Y_C_*’’*, Z_C_*’’), respectively. So, the origin (o_coil_’’) and three axes (x_coil_’’, y_coil_’’, and z_coil_’’) of the sphere-determined coordinate system on coil C_c_’’ could be defined with a 4 × 4 pose matrix and expressed as follows:

$C_{c}’’=\left[ x_{\mathrm{coil}}’’,y_{\mathrm{coil}}’’,z_{\mathrm{coil}}’’,o_{\mathrm{coil}}’’ \right]^{\perp},$ (S12)

where

$$\begin{matrix} \begin{matrix} o_{coil}’’=\left( X_{A}’’,Y_{A}’’,Z_{A}’’,1 \right)^{T}; \\ x_{coil}’’=\vec{A’’B’’}=\left( X_{B}’’-X_{A}’’,{Y_{B}’’-Y}_{A}’’,{Z_{B}’’-Z}_{A}’’,0 \right)^{T}; \end{matrix} \\ \begin{matrix} y_{coil}’’=\vec{A’’C’’}=\left( X_{C}’’-X_{A}’’,{Y_{C}’’-Y}_{A}’’,{Z_{C}’’-Z}_{A}’’,0 \right)^{T}; \\ z_{coil}’’={(\vec{A’’B’’}\times\vec{A’’C’’})}^{T}. \end{matrix} \end{matrix}$$

The coordinates of the centers of three spheres (D’’, E’’, F’’) on the subject’s head were defined as (*X_D_*’’*, Y_D_*’’*, Z_D_*’’), (*X_E_*’’*, Y_E_*’’*, Z_E_*’’), and (*X_F_*’’*, Y_F_*’’*, Z_F_*’’), respectively. So, the origin (*o_head_*’’) and three axes (*x_head_*’’, *y_head_*’’, and *z_head_*’’) of the sphere-determined coordinate system on the subject’s head C_s_’’ could be defined with a 4 × 4 pose matrix and expressed as follows:

$C_{s}’’=\left[ x_{\mathrm{head}}’’,y_{\mathrm{head}}’’,z_{\mathrm{head}}’’,o_{\mathrm{head}}’’ \right]^{\perp},$ (S13)

where

$$\begin{matrix} \begin{matrix} o_{\mathrm{head}}’’=\left( X_{D}’’,Y_{D}’’,Z_{D}’’,1 \right)^{T}; \\ x_{\mathrm{head}}’’=\vec{D’’E’’}=\left( X_{E}’’-X_{D}’’,{Y_{E}’’-Y}_{D}’’,{Z_{E}’’-Z}_{D}’’,0 \right)^{T}; \end{matrix} \\ \begin{matrix} y_{\mathrm{head}}’’=\vec{D’’F’’}=\left( X_{F}’’-X_{D}’’,{Y_{F}’’-Y}_{D}’’,{Z_{F}’’-Z}_{D}’’,0 \right)^{T}; \\ z_{\mathrm{head}}’’={(\vec{D’’E’’}\times\vec{D’’F’’})}^{T}. \end{matrix} \end{matrix}$$

Then, as shown in Figure 1A(11), the transformation T6 from C_s_’’ to C_c_’’ in laser-scan space could be expressed as follows:

$T6={C_{s}’’}^{-1}*C_{c}’’.$ (S14)

Finally, the transformation T2, which is shown in Figure 1A(13), from C_s_’’ to the planned coil position in 3D laser scan space could be expressed as follows:

$T2=T6*T3.$ (S15)

T2 represents the detected transformation from the sphere-determined coordinate system on the subject’s head to the planned coil position when stimulating the setup target in real coil positioning scenarios.

Owing to the error of installation and mechanical matching error between the 3D-printed models of the helmet, the subject’s head, and the coil, T cannot be equal to T2. The coil position error could be expressed as follows:

$E_{\mathrm{trans}}=\sqrt{{(T_{1,4}-{T2}_{1,4})}^{2}+{(T_{2,4}-{T2}_{2,4})}^{2}+{(T_{3,4}-{T2}_{3,4})}^{2}}$. (S16)

The coil orientation error could be expressed as follows:

$E_{\emptyset}=\left| \emptyset\right|$, (S17)

where (k, $\emptyset$) is the axis-angle representation of the rotation matrix of A’, which is defined as follows:

$A’=T*{T2}^{-1}$. (S18)

1. **Construction of phantom model for simulating soft skin and hair**

As shown in Figure 1B, the 3D model of the subject's skull was segmented and reconstructed from structural MRI data. Then, the 3D models of the subject’s head and skull were cut into upper and lower parts along the eyebrow arch. Next, the lower part of the subject’ head and the upper part of the subject’s skull were subjected to a Boolean operation of sum to create a skull model. Simultaneously, the upper part of the subject’ head and the upper part of the subject’s skull were subjected to a Boolean operation of difference to create a skin model. The skull model was 3D printed with hard resin materials, and the skin model was 3D printed using soft silicone material to simulate skin. Then, the skin model was stretched over the skull model to create a test model with soft skin. Finally, the wig was put on the skin model to test the influence of hair on the coil positioning accuracy of I-Helmet. Besides, as shown in Figure 1A(5), a completely hard model was 3D-printed for comparison.

In the present study, for each subject, three soft skin models were 3D printed using silica gel with Shore hardness values of 40, 50, and 60 [4-6]. To test the influence of different hair styles on coil positioning accuracy, three different wig styles (wig1: short straight hair [ZM011-1], wig2: short curly hair [Z651], wig3: long straight hair [Z576], Bestung, Jinhua, China) were applied.

1. **3D printing and model selection**

All models were printed using a 3D printer (Lite 600, UnionTech, Shanghai, China) with resin (UTR9400) with a layer thickness of 0.05 mm. Skin models were made using the reverse die method with silica gel (Hei-Cast 8158, H&K Ltd., Tokyo, Japan). To avoid possible warping and other problems in 3D printing, after the model 3D-printing was completed, the 3D-printed model was pre-scanned with the 3D scanner to obtain the coordinates of the centers of six spheres (A’’’, B’’’, C’’’, D’’’, E’’’, F’’’) and to carry out subsequent experiments on the models. The merged 3D model must satisfy both Eq. (S19) and Eq. (S20) simultaneously. The coil model must satisfy Eq. (S19), and the head model must satisfy Eq. (S20).

$\begin{matrix} \sqrt{\left( X_{A}’’’-X_{B}’’’ \right)^{2}+\left( Y_{A}’’’-Y_{B}’’’ \right)^{2}+\left( Z_{A}’’’-Z_{B}’’’ \right)^{2}}-50 \mathrm{mm}<0.3 \mathrm{mm} \\ \begin{matrix} \mathrm{and} \\ \sqrt{\left( X_{A}’’’-X_{C}’’’ \right)^{2}+\left( Y_{A}’’’-Y_{C}’’’ \right)^{2}+\left( Z_{A}’’’-Z_{C}’’’ \right)^{2}}-50 \mathrm{mm}<0.3 mm; \end{matrix} \end{matrix}$ (S19)

$\begin{matrix} \begin{matrix} \begin{matrix} \sqrt{\left( X_{D}’’’-X_{E}’’’ \right)^{2}+\left( Y_{D}’’’-Y_{E}’’’ \right)^{2}+\left( Z_{D}’’’-Z_{E}’’’ \right)^{2}}-70 \mathrm{mm}<0.3 \mathrm{mm} \\ \mathrm{and} \end{matrix} \end{matrix} \\ \sqrt{\left( X_{D}’’’-X_{F}’’’ \right)^{2}+\left( Y_{D}’’’-Y_{F}’’’ \right)^{2}+\left( Z_{D}’’’-Z_{F}’’’ \right)^{2}}-50 \mathrm{mm}<0.3 mm; \end{matrix}$ (S20)

where (*X_A_*’’’*, Y_A_*’’’*, Z_A_*’’’), (*X_B_*’’’*, Y_B_*’’’*, Z_B_*’’’), (*X_C_*’’’*, Y_C_*’’’*, Z_C_*’’’), (*X_D_*’’’*, Y_D_*’’’*, Z_D_*’’’), (*X_E_*’’’*, Y_E_*’’’*, Z_E_*’’’), and (*X_F_*’’’*, Y_F_*’’’*, Z_F_*’’’) are the centers of six spheres (A’’’, B’’’, C’’’, D’’’, E’’’, F’’’), respectively.

1. **Testing the influences of skin and hair on coil positioning accuracy of I-Helmet**

To test the influences of skin and hair on coil positioning accuracy, a hard resin model (Figure 1A (5)), a soft skin model (Figure 1B (11), 50-hardness), and a hair model (Figure 1B (12), wig1, 50-hardness skin) were applied to calculate the coil position and orientation error. A helmet with 0% tolerance was applied for coil guidance. After positioning the coil 3D model on the phantom model with the guidance of helmets, a 3D laser scan was applied to determine the centers of the six spheres in the experimental scenario. Then, Eq. (S16) and Eq. (S17) were applied to calculate the coil position and orientation error with the hard resin model, soft skin model, and hair model.

1. **Testing the influences of tolerance on the contact force and coil positioning accuracy**

To test the influences of tolerance on the head contact force and coil positioning accuracy, the hair 3D model (wig1, 50-hardness skin) was included for this experiment. As shown in Supplementary Figure 2A and B, eight membrane force sensors were stuck outside the skin of the model, and the contact forces were measured with a data acquisition card (USB-1208LS, ChengTEC, Shanghai). All force sensors were about 10 mm above the lower edge of the helmet. After the wig was put on the model, 20 readings of eight force sensors were recorded and averaged as the baseline of contact force. The baseline and helmets with tolerances of 3%, 4%, and 5% were evaluated with DF-9-40-20N (ChengTEC, Shanghai, China) for lower detection threshold (0.2 N), and helmets with tolerances of 0%, 1%, and 2% were evaluated with DF-9-40-50N (ChengTEC, Shanghai, China) for higher detection range. Then, six helmets with tolerances of 0%–5% were successively put on the hair 3D model. The 3D laser scanner was used to determine the centers of the six spheres in the experimental scenario. Then, Eq. (S16) and Eq. (S17) were applied to calculate the coil position and orientation errors corresponding to the six different helmet tolerances. At the same time, 20 readings of the eight force sensors were recorded and averaged, and then, the baseline was subtracted from the average to provide the contact force data between the helmet and the subject's head.


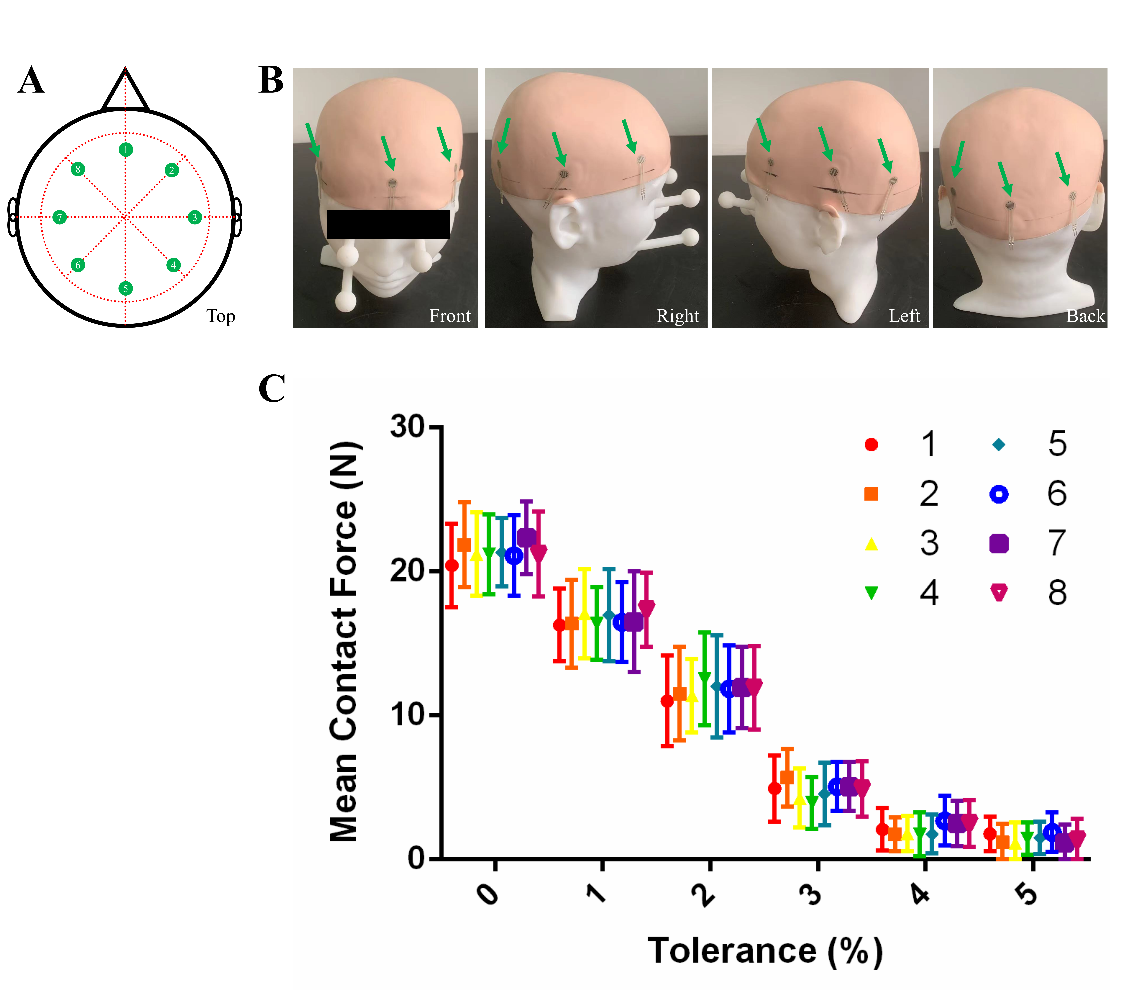


**Supplementary Figure 2. (A)** Diagram of top view of the distribution of eight membrane force sensors. **(B)** Photograph of the actual distribution of eight membrane force sensors on the phantom. **(C)** Effect of helmet tolerance on contact force.

1. **Comparing the effects of using helmet with or without landmark on coil positioning accuracy**

The hair model (wig1, 50-hardness skin) was included for this experiment. For each subject, landmark sticks were created for the 3%, 4%, and 5% tolerance helmets. First, the coil model was positioned on the hair model guided by the helmet without landmark sticks. Then, as shown in Figure 4A, the operator placed the helmet on the hair model with the guidance of the landmark sticks and positioned the coil model. The application scenario of the landmark guided helmet is shown in Figure 4B. Finally, the 3D laser scanner was applied to determine the centers of the six spheres in the experimental scenario with and without landmarks. Then, Eq. (S16) and Eq. (S17) were applied to calculate the coil position and orientation errors.

1. **Comparing the effects of different skin hardnesses, wig styles, and operators on coil positioning accuracy**

Three hair models with wig1 (40-hardness skin, 50-hardness skin, and 60-hardness skin) were included to test the effects of different skin hardnesses on coil positioning accuracy. Three hair models with 50-hardness skin (wig1, wig2, and wig3) were introduced to test the effects of different wig styles on coil positioning accuracy, as shown in supplementary Figure 3. Another two operators (one male and one females, aged 30–35 years) were introduced to test whether the operators could master the operation of I-Helmet within a short period of training time. The other two operators did not have any operating experience related to TMS or neuro-navigation, and they only had about 3 min of explanation and 5 min of practice before the real experiment. A hair model (wig1, 50-hardness skin) was applied for testing the effects of different operators on coil positioning accuracy. The landmark guided helmet with 3% tolerance was applied for testing the coil positioning accuracy in this section. Finally, the 3D laser scanner was applied to determine the centers of the six spheres in the experimental scenario. Then, Eq. (S16) and Eq. (S17) were applied to calculate the coil position and orientation errors.


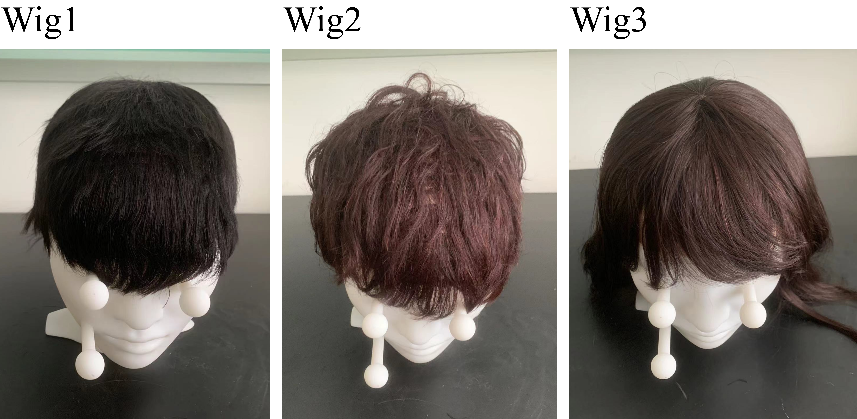


**Supplementary Figure 3.** Three wig styles applied in this study.

1. **Data analysis**

Linear regression was performed on the mean detected position and orientation errors and the manually set position and orientation errors.

One-way analysis of variance (ANOVA) was used to analyze the influences of skin and hair on coil positioning accuracy. Factors considered in the ANOVA were the three different models (hard model, skin model, and hair model).

One-way ANOVA was used to analyze the influence of tolerance on the coil positioning accuracy. Factors considered in the ANOVA were the six different tolerances (from 0% to 5% in 1% increments). Two-way ANOVA was used to analyze the influence of tolerance on the contact force. Factors considered in the ANOVA were the eight sensor positions (shown in Supplementary Figure 4A) and six tolerances (from 0% to 5% in 1% increments).

Paired T-tests were applied to compare the effects of using the helmets with or without landmarks on the coil positioning accuracy. One-way ANOVA was used to compare the effects of different skin hardnesses, wig styles, and operators on the coil positioning accuracy.

All data analyses were performed with Prism 6 software (GraphPad Inc., La Jolla, CA, USA).


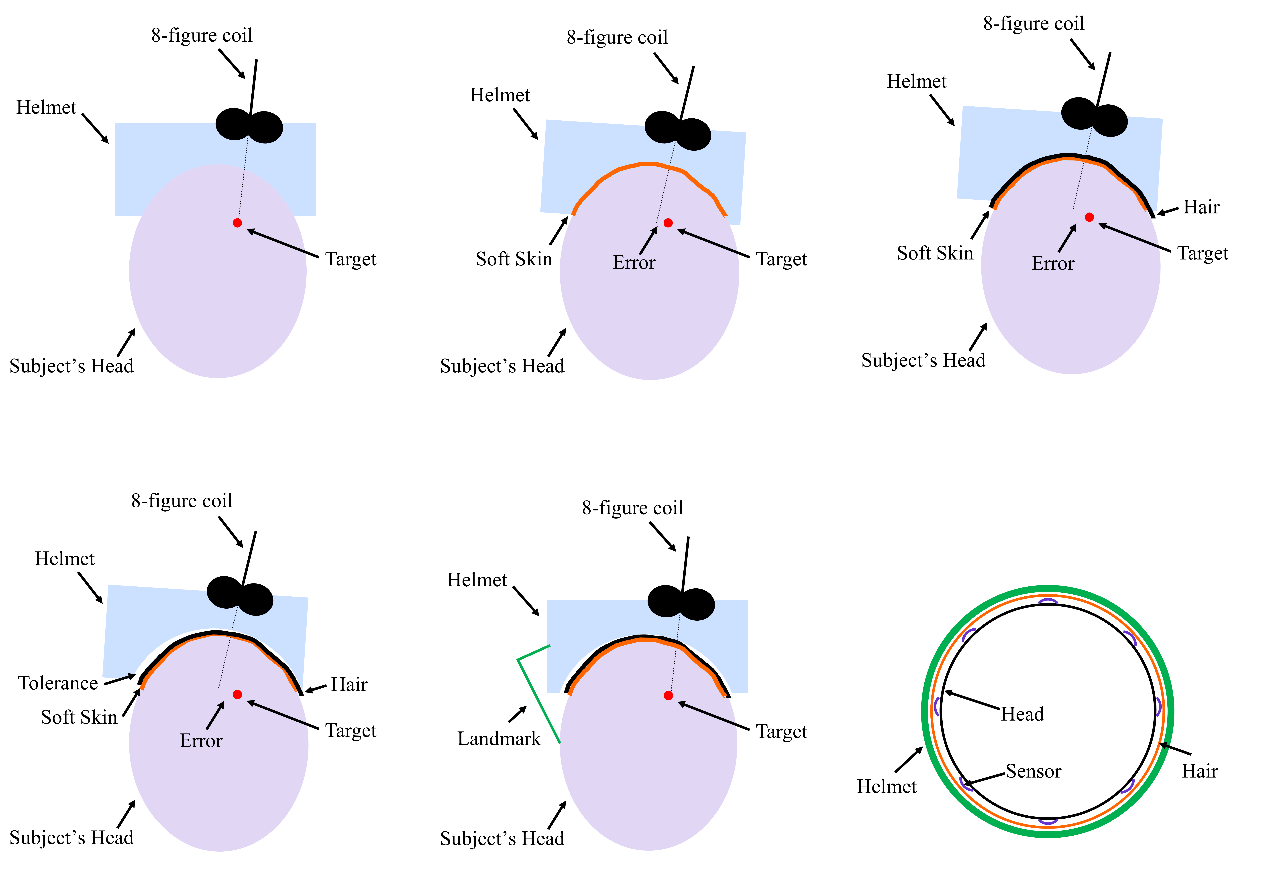


**Supplementary Figure 4.** Schematic diagram for explaining the experimental results in this study.

Supplementary Table2. The influences of skin and hair on the coil positioning accuracy of I-Helmet

|  | Position Error (mm) | | | Orientation Error (°) | | |
| --- | --- | --- | --- | --- | --- | --- |
|  | Hard | Skin | Hair | Hard | Skin | Hair |
| Subject1 | 1.922635 | 2.811253 | 3.879367 | 2.843585 | 2.448851 | 3.648758 |
| Subject2 | 2.521138 | 3.090869 | 4.223160 | 2.347082 | 2.971152 | 4.196354 |
| Subject3 | 2.003087 | 2.683955 | 3.635505 | 2.039961 | 2.84046 | 4.142418 |
| Subject4 | 2.038266 | 3.329064 | 3.961820 | 2.959771 | 3.201442 | 3.403739 |
| Subject5 | 2.002952 | 2.948614 | 3.821895 | 1.983569 | 2.654268 | 3.620969 |
| Subject6 | 2.215049 | 2.079541 | 3.775167 | 2.083862 | 2.204665 | 4.019954 |
| Subject7 | 1.968323 | 2.888443 | 4.112851 | 2.151796 | 2.693436 | 3.960557 |
| Subject8 | 1.825177 | 2.856491 | 4.342912 | 2.451738 | 3.03354 | 3.826077 |
| Subject9 | 1.913649 | 3.182853 | 4.439394 | 2.253322 | 2.751314 | 4.450005 |
| Subject10 | 2.165011 | 3.081668 | 4.238076 | 2.710524 | 3.029289 | 4.572506 |
| Subject11 | 1.790508 | 1.843890 | 3.973037 | 1.936747 | 1.716991 | 3.625812 |
| Subject12 | 1.139879 | 2.966035 | 4.206020 | 1.703597 | 2.650988 | 3.645924 |
| Subject13 | 1.870759 | 3.584712 | 3.662415 | 2.021017 | 3.427984 | 4.138727 |
| Subject14 | 2.767265 | 2.551626 | 4.295065 | 2.828996 | 2.777058 | 4.123379 |
| Subject15 | 1.778336 | 2.496301 | 4.759065 | 1.774738 | 2.284609 | 3.842266 |
| Subject16 | 2.321577 | 2.690369 | 3.131991 | 2.06569 | 2.70126 | 3.30594 |
| Subject17 | 2.672556 | 2.152185 | 4.495146 | 1.967817 | 3.040259 | 4.229547 |
| Subject18 | 1.839839 | 2.500491 | 4.194889 | 1.570306 | 2.291658 | 3.733513 |
| Subject19 | 2.006852 | 2.057532 | 4.456818 | 1.652035 | 2.627358 | 3.643486 |
| Subject20 | 1.949594 | 2.848576 | 3.593723 | 1.699767 | 2.365599 | 4.061704 |
| Subject21 | 1.760290 | 3.010825 | 4.369422 | 1.924813 | 3.333855 | 4.42481 |
| Subject22 | 1.443000 | 3.111881 | 4.526587 | 1.45893 | 3.160386 | 3.506412 |
| Subject23 | 2.307867 | 1.599343 | 4.087259 | 1.957167 | 1.606321 | 3.851992 |
| Subject24 | 1.878636 | 2.687874 | 4.212696 | 2.166421 | 3.120593 | 4.035659 |
| Mean | 2.00 | 2.71 | 4.10 | 2.11 | 2.71 | 3.92 |
| STD | 0.35 | 0.48 | 0.37 | 0.41 | 0.46 | 0.34 |

Supplementary Table 3 The influences of helmet tolerance on the coil positioning erro of I-Helmet

|  | Position Error (mm) | | | | | |
| --- | --- | --- | --- | --- | --- | --- |
| Tolerance | 0% | 1% | 2% | 3% | 4% | 5% |
| Subject1 | 3.983176 | 4.2889 | 5.373463 | 5.695767 | 6.281121 | 7.179726 |
| Subject2 | 3.938529 | 4.587764 | 4.891416 | 6.030288 | 6.685137 | 7.34579 |
| Subject3 | 4.419832 | 4.77294 | 4.942597 | 5.471057 | 5.429366 | 6.106178 |
| Subject4 | 3.825375 | 3.708523 | 4.711069 | 6.03331 | 6.465335 | 6.641179 |
| Subject5 | 4.330441 | 3.998919 | 5.765109 | 6.393216 | 6.898811 | 7.600667 |
| Subject6 | 4.222977 | 3.735831 | 5.314765 | 5.72231 | 6.652167 | 7.240447 |
| Subject7 | 4.179611 | 3.632188 | 5.244214 | 6.274647 | 6.893658 | 7.855252 |
| Subject8 | 3.725799 | 3.788155 | 4.414884 | 6.453241 | 7.46019 | 8.107191 |
| Subject9 | 3.673301 | 4.589644 | 4.683259 | 6.310515 | 6.286967 | 7.791435 |
| Subject10 | 4.316365 | 4.824492 | 4.856988 | 4.589422 | 7.035162 | 6.651133 |
| Subject11 | 4.329841 | 4.475888 | 5.309265 | 5.415374 | 6.285631 | 6.597755 |
| Subject12 | 3.577233 | 4.526917 | 5.60284 | 5.465906 | 8.203162 | 8.695711 |
| Subject13 | 4.535769 | 4.372902 | 5.447782 | 5.494104 | 6.92415 | 8.48518 |
| Subject14 | 3.889489 | 4.447443 | 5.606631 | 5.438118 | 6.813516 | 6.894676 |
| Subject15 | 4.300315 | 3.917867 | 5.550106 | 4.850808 | 9.059737 | 7.536668 |
| Subject16 | 3.077115 | 2.976668 | 5.885094 | 5.171635 | 7.669092 | 9.099172 |
| Subject17 | 4.187394 | 5.177613 | 6.447544 | 7.262145 | 8.137471 | 8.183058 |
| Subject18 | 4.099458 | 4.894262 | 6.147712 | 5.581048 | 7.283263 | 8.362517 |
| Subject19 | 4.472966 | 3.739037 | 5.30595 | 6.169241 | 9.42473 | 8.884583 |
| Subject20 | 3.591018 | 3.828079 | 4.572343 | 6.098231 | 7.234945 | 8.500691 |
| Subject21 | 2.914539 | 5.336437 | 4.954676 | 5.95769 | 7.49774 | 7.367808 |
| Subject22 | 3.949456 | 3.929148 | 5.109756 | 6.574953 | 7.416421 | 8.421935 |
| Subject23 | 4.247991 | 3.820265 | 4.852742 | 4.990702 | 7.596218 | 8.236912 |
| Subject24 | 4.459614 | 5.252122 | 5.19614 | 6.520882 | 7.594809 | 7.109739 |
| Mean | 4.01 | 4.28 | 5.26 | 5.83 | 7.22 | 7.70 |
| STD | 0.42 | 0.59 | 0.50 | 0.62 | 0.89 | 0.81 |
|  | Rotation Error (°) | | | | | |
| Tolerance | 0% | 1% | 2% | 3% | 4% | 5% |
| Subject1 | 3.254255 | 4.134071 | 5.465662 | 5.506055 | 6.099278 | 6.672776 |
| Subject2 | 4.208918 | 4.269367 | 5.086915 | 6.250618 | 6.925117 | 7.210122 |
| Subject3 | 3.689163 | 4.444087 | 4.64811 | 5.517074 | 5.593553 | 6.061986 |
| Subject4 | 3.984209 | 4.396327 | 4.837108 | 4.382385 | 6.15003 | 5.328701 |
| Subject5 | 4.347286 | 4.545208 | 5.555352 | 6.173159 | 6.635703 | 7.541544 |
| Subject6 | 4.132237 | 4.423448 | 4.848971 | 5.287303 | 6.746702 | 7.323407 |
| Subject7 | 3.989383 | 4.552045 | 4.699236 | 6.252729 | 6.410371 | 7.246318 |
| Subject8 | 3.667119 | 4.580527 | 4.9323 | 5.833974 | 6.703257 | 8.1638 |
| Subject9 | 4.490641 | 4.898022 | 5.332689 | 6.783198 | 6.660468 | 7.605693 |
| Subject10 | 4.184677 | 4.788438 | 4.542831 | 5.578609 | 7.118701 | 7.244382 |
| Subject11 | 4.052812 | 3.928018 | 4.856499 | 5.001508 | 6.389139 | 6.703701 |
| Subject12 | 4.535651 | 4.405394 | 4.592077 | 5.334552 | 7.127108 | 8.552525 |
| Subject13 | 2.503684 | 4.786102 | 5.356219 | 4.927996 | 7.493 | 9.001255 |
| Subject14 | 4.236826 | 4.524169 | 5.187307 | 5.254843 | 7.882775 | 6.164949 |
| Subject15 | 3.228101 | 4.423607 | 6.423245 | 6.487737 | 7.340046 | 7.880528 |
| Subject16 | 4.426329 | 4.031736 | 5.997739 | 5.820156 | 7.227146 | 6.285871 |
| Subject17 | 3.951691 | 6.033773 | 6.381211 | 6.66214 | 6.544358 | 7.137092 |
| Subject18 | 4.319248 | 4.587283 | 5.515603 | 5.860609 | 6.705974 | 7.195116 |
| Subject19 | 4.212753 | 4.91514 | 5.748572 | 6.488947 | 7.238825 | 7.582136 |
| Subject20 | 3.787974 | 4.987568 | 5.66026 | 5.796201 | 7.446824 | 6.904514 |
| Subject21 | 3.906258 | 4.034737 | 5.299246 | 6.13112 | 6.95205 | 9.308323 |
| Subject22 | 4.082887 | 3.998024 | 5.895931 | 6.268876 | 8.209304 | 7.983284 |
| Subject23 | 4.040571 | 4.272481 | 5.904478 | 6.376154 | 7.265359 | 8.595681 |
| Subject24 | 3.66035 | 5.076657 | 5.174511 | 6.460695 | 7.613386 | 8.420902 |
|  | 3.95 | 4.54 | 5.33 | 5.85 | 6.94 | 7.42 |
|  | 0.46 | 0.45 | 0.54 | 0.61 | 0.60 | 0.97 |

Supplementary Table 4 Effects of using helmet with or without landmarks on coil positioning accuracy

|  | Position Error (mm) | | | | | |
| --- | --- | --- | --- | --- | --- | --- |
| Tolerance | 3% | | 4% | | 5% | |
| LandMark | With | Without | With | Without | With | Without |
| Subject1 | 4.609874 | 5.933273 | 4.540086 | 5.995982 | 3.956121 | 6.135907 |
| Subject2 | 4.564458 | 5.634028 | 4.251759 | 5.439416 | 4.712218 | 7.000076 |
| Subject3 | 3.900109 | 5.475884 | 3.741768 | 6.077116 | 3.769416 | 5.983407 |
| Subject4 | 3.477204 | 6.179973 | 3.814139 | 8.917829 | 4.046338 | 8.115902 |
| Subject5 | 4.058322 | 7.017977 | 3.860345 | 7.097451 | 4.142661 | 7.333067 |
| Subject6 | 3.591845 | 6.490001 | 3.895983 | 7.978468 | 3.854251 | 7.438496 |
| Subject7 | 3.866796 | 6.014051 | 4.487837 | 6.297106 | 4.378488 | 7.751721 |
| Subject8 | 3.834103 | 6.148246 | 4.258913 | 6.072969 | 4.586971 | 6.292776 |
| Subject9 | 4.413918 | 6.275635 | 3.824284 | 6.602859 | 3.860398 | 6.884975 |
| Subject10 | 3.414629 | 5.899478 | 4.115318 | 6.7131 | 4.355785 | 7.646227 |
| Subject11 | 4.058693 | 6.111507 | 4.682264 | 7.887738 | 4.305555 | 8.287768 |
| Subject12 | 3.16737 | 5.118029 | 4.486977 | 6.99295 | 4.376338 | 7.966709 |
| Subject13 | 3.378581 | 6.906118 | 4.873375 | 7.009583 | 3.131049 | 8.090693 |
| Subject14 | 3.570989 | 5.578998 | 3.667166 | 8.04729 | 4.357974 | 7.10937 |
| Subject15 | 4.161527 | 6.281419 | 4.016598 | 7.715831 | 4.360276 | 8.173414 |
| Subject16 | 4.095746 | 6.514413 | 3.97508 | 6.822284 | 4.375499 | 8.265577 |
| Subject17 | 4.476817 | 6.394853 | 5.84301 | 7.385741 | 4.161186 | 7.458831 |
| Subject18 | 3.123817 | 6.50061 | 4.868942 | 6.556759 | 5.000983 | 7.549924 |
| Subject19 | 3.362332 | 6.419865 | 5.104097 | 7.963337 | 4.347505 | 7.383157 |
| Subject20 | 3.935178 | 5.94055 | 4.925169 | 6.907699 | 5.462789 | 8.351063 |
| Subject21 | 4.135281 | 7.727913 | 4.473986 | 7.274981 | 5.688073 | 7.999136 |
| Subject22 | 3.578156 | 7.359719 | 4.085568 | 6.895687 | 4.667329 | 7.577907 |
| Subject23 | 4.457158 | 5.617891 | 4.947728 | 7.228158 | 4.382958 | 8.075777 |
| Subject24 | 3.785546 | 6.843344 | 4.514936 | 7.868791 | 4.203909 | 7.745923 |
| Mean | 3.88 | 6.27 | 4.39 | 7.07 | 4.35 | 7.53 |
| STD | 0.44 | 0.61 | 0.53 | 0.81 | 0.53 | 0.68 |
|  | Rotation Error (°) | | | | | |
| Tolerance | 3% | | 4% | | 5% | |
| LandMark | After | Before | After | Before | After | Before |
| Subject1 | 4.202223 | 5.649098 | 4.122342 | 5.954982 | 4.284583 | 6.58836 |
| Subject2 | 4.358115 | 5.658968 | 4.220447 | 6.306727 | 5.038312 | 6.615684 |
| Subject3 | 3.549339 | 5.412086 | 3.374937 | 5.828631 | 4.046315 | 5.682239 |
| Subject4 | 3.576732 | 5.874174 | 3.938649 | 7.240195 | 5.405213 | 7.272564 |
| Subject5 | 4.051692 | 5.823903 | 3.715438 | 6.69069 | 3.450509 | 6.414652 |
| Subject6 | 3.772076 | 5.697628 | 4.057395 | 7.539733 | 3.90384 | 7.033088 |
| Subject7 | 4.020718 | 5.243243 | 4.066331 | 6.929309 | 3.634316 | 7.071548 |
| Subject8 | 4.300102 | 6.365265 | 4.44953 | 6.941106 | 4.586726 | 7.544117 |
| Subject9 | 3.411352 | 6.154568 | 3.685223 | 5.453053 | 4.283864 | 7.332953 |
| Subject10 | 4.46404 | 6.356892 | 3.954501 | 6.46784 | 4.069106 | 6.918053 |
| Subject11 | 3.826177 | 5.66005 | 4.039106 | 6.60986 | 4.139393 | 7.057796 |
| Subject12 | 4.053073 | 5.874861 | 3.999246 | 6.68802 | 3.39807 | 7.159914 |
| Subject13 | 4.125795 | 6.23622 | 4.042514 | 7.165049 | 3.489113 | 8.412947 |
| Subject14 | 4.435744 | 5.376548 | 4.099638 | 6.940602 | 4.309603 | 6.858941 |
| Subject15 | 3.795767 | 6.261892 | 3.817502 | 7.050949 | 4.483827 | 7.628454 |
| Subject16 | 4.063743 | 6.167241 | 3.759907 | 7.363784 | 4.106168 | 8.133994 |
| Subject17 | 4.224348 | 5.475834 | 3.589153 | 6.5624 | 5.058413 | 7.115008 |
| Subject18 | 3.313962 | 5.494423 | 4.094237 | 7.521524 | 4.334269 | 7.873537 |
| Subject19 | 4.101342 | 6.717143 | 4.27219 | 6.871434 | 4.186959 | 6.928192 |
| Subject20 | 3.64171 | 5.294736 | 4.6076 | 7.237264 | 4.670338 | 7.248995 |
| Subject21 | 3.963466 | 6.882792 | 4.608865 | 7.298737 | 4.75514 | 7.834333 |
| Subject22 | 4.142522 | 6.894055 | 3.521957 | 7.042414 | 4.112754 | 6.450829 |
| Subject23 | 4.110519 | 6.958966 | 4.143642 | 7.297435 | 4.481223 | 7.975844 |
| Subject24 | 4.403541 | 6.56024 | 4.345558 | 7.637858 | 3.904781 | 8.781909 |
| Mean | 4.00 | 6.00 | 4.02 | 6.86 | 4.29 | 7.25 |
| STD | 0.32 | 0.54 | 0.32 | 0.56 | 0.51 | 0.70 |

Supplementary Table 5. Effects of different skin hardnesses, wig styles, and operators on coil positioning accuracy of landmark guided I-Helmet

|  | Position Error (mm) | | | | | |
| --- | --- | --- | --- | --- | --- | --- |
|  | wig1 | wig2 | Skin40 | Skin60 | Operator2 | Operator3 |
| Subject1 | 4.239928 | 4.133032 | 4.246642 | 4.207914 | 4.201214 | 4.422025 |
| Subject2 | 4.462363 | 3.888728 | 4.747206 | 4.853063 | 3.739656 | 4.988135 |
| Subject3 | 3.972753 | 4.411936 | 4.145695 | 3.959306 | 3.945245 | 4.242066 |
| Subject4 | 3.476531 | 4.291619 | 4.649276 | 4.070483 | 3.854286 | 4.088761 |
| Subject5 | 4.022556 | 3.823811 | 4.174726 | 4.408853 | 4.32838 | 4.340871 |
| Subject6 | 4.327815 | 3.960162 | 4.287268 | 3.905279 | 3.98571 | 4.238563 |
| Subject7 | 3.952811 | 3.64081 | 3.842956 | 4.471188 | 4.106322 | 4.142879 |
| Subject8 | 4.359889 | 3.446385 | 4.197082 | 3.496769 | 3.662275 | 3.916262 |
| Subject9 | 3.883037 | 3.413694 | 3.673768 | 4.410525 | 4.334497 | 4.06262 |
| Subject10 | 4.046915 | 4.077191 | 3.882511 | 4.241782 | 4.114124 | 3.82156 |
| Subject11 | 4.762631 | 4.02358 | 4.057098 | 4.757178 | 4.037759 | 4.225717 |
| Subject12 | 3.304179 | 3.784309 | 3.938459 | 3.547117 | 3.866604 | 3.28362 |
| Subject13 | 3.277726 | 4.176596 | 4.001161 | 3.346255 | 4.524673 | 4.428354 |
| Subject14 | 4.366151 | 5.196197 | 4.129195 | 4.413985 | 4.136791 | 4.149463 |
| Subject15 | 3.995008 | 4.212129 | 2.471543 | 3.254093 | 4.543194 | 3.987244 |
| Subject16 | 4.677084 | 3.156018 | 4.222401 | 3.53145 | 3.891921 | 3.52745 |
| Subject17 | 4.568771 | 3.50763 | 2.978402 | 4.120795 | 3.811111 | 3.885354 |
| Subject18 | 4.074964 | 4.05327 | 4.954826 | 4.289215 | 4.361638 | 3.810186 |
| Subject19 | 4.218703 | 3.531567 | 4.680823 | 4.354615 | 3.61509 | 3.181337 |
| Subject20 | 4.52797 | 3.245748 | 5.08601 | 3.456996 | 3.502319 | 4.070884 |
| Subject21 | 4.663631 | 2.958598 | 3.706737 | 3.770378 | 4.093955 | 3.63192 |
| Subject22 | 4.478288 | 4.260274 | 4.105933 | 3.822132 | 4.080684 | 3.738452 |
| Subject23 | 4.978703 | 3.013938 | 4.850949 | 4.623145 | 4.133581 | 3.481137 |
| Subject24 | 3.583412 | 4.132084 | 3.41757 | 2.483213 | 4.939075 | 3.633448 |
| Mean | 4.18 | 3.85 | 4.10 | 3.99 | 4.08 | 3.97 |
| STD | 0.45 | 0.51 | 0.60 | 0.56 | 0.33 | 0.40 |
|  | Orientation Error (°) | | | | | |
|  | Wig2 | Wig3 | Skin40 | Skin60 | Operator2 | Operator3 |
| Subject1 | 4.663827 | 4.500843 | 4.603076 | 4.128538 | 4.147639 | 4.322928 |
| Subject2 | 4.607291 | 5.040868 | 4.010031 | 4.818546 | 4.297053 | 4.421304 |
| Subject3 | 3.814377 | 4.282373 | 4.477261 | 3.827776 | 4.104113 | 4.542169 |
| Subject4 | 3.445343 | 3.961167 | 3.14307 | 3.851852 | 4.257935 | 4.491511 |
| Subject5 | 3.5338 | 3.8531 | 4.242967 | 4.138233 | 4.221176 | 4.749149 |
| Subject6 | 4.407392 | 3.537041 | 4.63754 | 3.714904 | 3.801091 | 4.056916 |
| Subject7 | 3.464865 | 3.900672 | 4.085847 | 4.471283 | 3.954276 | 4.481615 |
| Subject8 | 4.370809 | 3.467373 | 4.169178 | 4.144888 | 4.031149 | 4.445414 |
| Subject9 | 4.262697 | 3.542137 | 4.315121 | 4.272126 | 3.511049 | 3.747201 |
| Subject10 | 4.370249 | 4.316893 | 4.416214 | 3.753657 | 4.223514 | 4.094183 |
| Subject11 | 4.506072 | 4.240702 | 3.984195 | 4.441386 | 3.965599 | 4.308297 |
| Subject12 | 3.817907 | 3.451657 | 3.772164 | 3.712412 | 3.554105 | 4.324346 |
| Subject13 | 4.068441 | 3.872899 | 4.080194 | 4.229573 | 3.829374 | 4.408687 |
| Subject14 | 3.522846 | 4.733797 | 3.605912 | 4.357065 | 4.076987 | 4.155421 |
| Subject15 | 4.570692 | 4.319055 | 3.98767 | 4.322975 | 4.099677 | 3.878574 |
| Subject16 | 3.840534 | 4.433648 | 4.313364 | 3.800299 | 3.866822 | 3.730619 |
| Subject17 | 3.831105 | 4.305384 | 4.532716 | 4.284662 | 4.313013 | 3.505386 |
| Subject18 | 4.366376 | 3.991296 | 4.703562 | 3.951083 | 3.957691 | 4.100108 |
| Subject19 | 4.472476 | 4.226575 | 3.828769 | 4.365281 | 4.269082 | 4.28153 |
| Subject20 | 4.358643 | 3.587308 | 3.803989 | 4.154932 | 4.09165 | 3.65107 |
| Subject21 | 3.939892 | 4.197595 | 3.550455 | 3.434596 | 4.193932 | 4.36403 |
| Subject22 | 3.910701 | 4.51382 | 4.516695 | 3.909559 | 3.759892 | 3.684387 |
| Subject23 | 3.545292 | 4.531993 | 4.030541 | 4.753511 | 4.440376 | 4.577121 |
| Subject24 | 4.441207 | 4.326512 | 4.004757 | 4.293757 | 4.086204 | 4.265939 |
| Mean | 4.09 | 4.13 | 4.12 | 4.13 | 4.04 | 4.19 |
| STD | 0.41 | 0.42 | 0.39 | 0.38 | 0.23 | 0.33 |

1. **Reference**

[1] He Wang, Jingna Jin, Xin Wang, Ying Li, Wenqing Liao, Dong Cui, Zhipeng Liu, Tao Yin, Individualized and clinically friendly helmet-type coil positioning method (I-Helmet) for transcranial magnetic stimulation. Brain Stimulation. 2022;15(5):1023-1025, <https://doi.org/10.1016/j.brs.2022.07.045>.

[2] He Wang, Jingna Jin, Xin Wang, Ying Li, Zhipeng Liu and Tao Yin. Non-orthogonal one-step calibration method for robotized transcranial magnetic stimulation. BioMed Eng OnLine. 2018;(17): 137. <https://doi.org/10.1186/s12938-018-0570-9>

[3] Lars R, Floris E, Alexer S, et al. Robust real-time robot-world calibration for robotized transcranial magnetic stimulation. Int J Med Robot. 2011;7:414

[4] Wang W H. SILICONE ELASTOMER COMPOSITION FOR ARTIFICIAL SKIN: WO, WO2008072517 A1[P].

[5] Kuang W , Lin D , Dacheng W U, Formulation of X-ray phantom materials[J]. Chinese Science Bulletin, 1995, 17:80-84.

[6] Zhang Y, Zheng J, Magnenat-Thalmann N. Example-guided anthropometric human body modeling[J]. Visual Computer, 2015, 31(12):1615-1631.
